# Ensemble bootstrap methodology for forecasting dynamic growth processes using differential equations: application to epidemic outbreaks

**DOI:** 10.1186/s12874-021-01226-9

**Published:** 2021-02-14

**Authors:** Gerardo Chowell, Ruiyan Luo

**Affiliations:** 1 Department of Population Heath Sciences, School of Public Health, Georgia State University, Atlanta, GA USA; 2 Division of International Epidemiology and Population Studies, Fogarty International Center, National Institutes of Health, Bethesda, MD USA

**Keywords:** Model ensemble, parameter estimation, uncertainty quantification, phenomenological growth, Differential equations, Generalized logistic growth model, Richards model, Gompertz model, Interval score, Parametric bootstrapping

## Abstract

**Background:**

Ensemble modeling aims to boost the forecasting performance by systematically integrating the predictive accuracy across individual models. Here we introduce a simple-yet-powerful ensemble methodology for forecasting the trajectory of dynamic growth processes that are defined by a system of non-linear differential equations with applications to infectious disease spread.

**Methods:**

We propose and assess the performance of two ensemble modeling schemes with different parametric bootstrapping procedures for trajectory forecasting and uncertainty quantification. Specifically, we conduct sequential probabilistic forecasts to evaluate their forecasting performance using simple dynamical growth models with good track records including the Richards model, the generalized-logistic growth model, and the Gompertz model. We first test and verify the functionality of the method using simulated data from phenomenological models and a mechanistic transmission model. Next, the performance of the method is demonstrated using a diversity of epidemic datasets including scenario outbreak data of the *Ebola Forecasting Challenge* and real-world epidemic data outbreaks of including influenza, plague, Zika, and COVID-19.

**Results:**

We found that the ensemble method that randomly selects a model from the set of individual models for each time point of the trajectory of the epidemic frequently outcompeted the individual models as well as an alternative ensemble method based on the weighted combination of the individual models and yields broader and more realistic uncertainty bounds for the trajectory envelope, achieving not only better coverage rate of the 95% prediction interval but also improved mean interval scores across a diversity of epidemic datasets.

**Conclusion:**

Our new methodology for ensemble forecasting outcompete component models and an alternative ensemble model that differ in how the variance is evaluated for the generation of the prediction intervals of the forecasts.

**Supplementary Information:**

The online version contains supplementary material available at 10.1186/s12874-021-01226-9.

## Introduction

The application of mathematical models to generate near real-time forecasts of the trajectory of epidemics and pandemics to guide public health interventions has been receiving increasing attention during the last decade. For instance, disease forecasting efforts have been conducted in the context of forecasting challenges such as the DARPA Chikungunya Challenge [1], the US CDC Flu sight Challenge [2], the Dengue Forecasting Challenge [3], and the Ebola Forecasting Challenge [4] as well as recent epidemic and pandemic emergencies including the 2014–16 West African Ebola epidemic [5, 6], the 2018–19 DRC Ebola epidemic [7] and the ongoing COVID-19 pandemic [8–12]. It is also worth noting that the diversity of mathematical models and approaches for epidemic forecasting has been expanding, with probabilistic forecasts gaining more attention [13, 14].

Assessing prediction accuracy is a key aspect of model-based forecasting especially in the context of limited epidemiological data or the emergence of novel pathogens for which little is known about the natural course of the disease. However, epidemiological data is frequently insufficient to discriminate among different plausible models. Hence, forecasting approaches that rely on multiple models rather than a single model are desirable [7, 15]. One powerful multi-model approach consists in devising ensemble models based on a quantitative combination of a set of individual models (e.g. [16–21]). While ensemble modeling has become a standard approach in weather forecasting systems [17, 18, 22–24], their application in infectious disease forecasting has only recently started to gain traction (e.g. [25–28]).

Ensemble modeling aims to boost the forecasting performance by systematically integrating the predictive accuracy tied to a set of individual models which can range from phenomenological, semi-mechanistic to fully mechanistic [16, 25, 29]. Past work indicates that multi-model ensemble approaches are powerful forecasting tools that frequently outperform individual models in epidemic forecasts [2–4, 7, 27, 30–32]. However, there is a lack of studies that systematically assess their forecasting performance across a diverse catalogue of epidemic datasets involving multiple infectious diseases and social contexts. In the context of influenza, one study utilized “weighted density ensembles” for predicting timing and severity metrics and found that the performance of the ensemble model was comparable to that of the top individual model albeit the ensemble’s forecasts were more stable across influenza seasons [33]. In the context of dengue in Puerto Rico, another study found that forecasts derived from Bayesian averaging ensembles outperformed a set of individual models [27]. Here we put forward and assess the performance of two frequentist computational ensemble modeling schemes for forecasting the trajectory of growth processes based on differential equations with applications to epidemic outbreaks [34]. For this purpose, we conduct sequential probabilistic forecasts to evaluate their forecasting performance using simple dynamical growth models with promising track records including the Richards model, the generalized-logistic growth model, and the Gompertz model and a diversity of epidemic datasets including synthetic data from standard epidemic models to demonstrate method functionality as well as scenario outbreak data of the *Ebola Forecasting Challenge* [4] and real epidemic data involving a range of infectious diseases including influenza, plague, Zika, and COVID-19.

### Parameter estimation for a given model

Given a model, parameter estimation is the process of finding the parameter values and their uncertainty that best explain empirical data. Here we briefly describe the parameter estimation method described in ref. [34] To calibrate dynamic models describing the trajectory of epidemics, temporal data for one or more states of the system (e.g., daily number of new outpatients, inpatients and deaths) are required. In this paper, if we consider the case with only one state of the system, we have:

$$ \dot{x}=g\left(x,\Theta \right) $$

Where $\dot{x}$ denotes the rate of change of the system and Θ = (*θ*_1_, *θ*_2_, …, *θ*_*m*_) is the set of model parameters. The temporal resolution of the data typically varies according to the time scale of the processes of interest (e.g, daily, weekly, yearly) and the frequency at which the state of the system is measured. We denote the time series of *n* longitudinal observations of the single state by:

$$ {y}_{t_j=}{y}_{t_1,}{y}_{t_2},\dots, {y}_{t_n}\ \mathrm{where}\ j=1,2,\dots, n $$

where *t*_*j*_ are the time points of the time series data and *n* is the number of observations. Let *f*(*t*, Θ) denote the expected incidence series *y*_*t*_ over time, which corresponds to $\dot{x}(t)$ if *x*(*t*) denotes the cumulative number of new cases at time *t*. Usually the incidence series ${y}_{t_j}$ is assumed to have a Poisson distribution with mean $\dot{x}(t)$ or a negative binomial distribution when the data exhibits overdispersion.

Model parameters are estimated by fitting the model solution to the observed data via nonlinear least squares [35] or via maximum likelihood estimation assuming a specific error structure in the data such as Poisson [36]. For nonlinear least squares, this is achieved by searching for the set of parameters $\hat{\varTheta}=\left({\hat{\theta}}_1,{\hat{\theta}}_2,\dots, {\hat{\theta}}_m\right)$ that minimizes the sum of squared differences between the observed data ${y}_{t_j=}{y}_{t_1,}{y}_{t_2}\dots ..{y}_{t_n}$ and the model mean which corresponds to *f*(*t*, Θ). That is, Θ = (*θ*_1_, *θ*_2_, …, *θ*_*m*_) is estimated by $\hat{\Theta}=\arg \min\ \sum \limits_{j=1}^n{\left(f\left({t}_j,\Theta \right)-{y}_{t_j}\right)}^2$.

Hence, the model mean $f\left(t,\hat{\Theta}\right)$ yields the best fit to the observed data in terms of squared L2 norm. This parameter estimation method gives the same weight to all of the data points, and does not require a specific distributional assumption for *y*_*t*_, except for the first moment *E*[*y*_*t*_] = *f*(*t*_*i*_; *Θ*); meaning, the mean at time *t* is equivalent to the count (e.g., number of cases) at time *t* [37]. Moreover, this method yields asymptotically unbiased point estimates regardless of any misspecification of the variance-covariance error structure. Hence, the model mean $f\left({t}_i,\hat{\Theta}\right)$ yields the best fit to observed data ${y}_{t_i}$ in terms of squared L2 norm.

The parameters for trajectories involving count data are often estimated via maximum likelihood estimation (MLE) with a Poisson error structure in the data. Consider the probability mass function (pmf) that specifies the probability of observing data *y*_*t*_ given the parameter set Θ, or *f*(*y*_*t*_| *Θ*); given a set of parameter values, the pmf can show which data are more probable, or more likely [37]. MLE aims to determine the values of the parameter set that maximizes the likelihood function, where the likelihood function is defined as *L*(*Θ*| *y*_*t*_) = *f*(*y*_*t*_| *Θ*) [37, 38]. The resulting parameter set is called the MLE estimate, the most likely to have generated the observed data. Specifically, the MLE estimate is obtained by maximizing the corresponding log-likelihood function. For count data with variability characterized by the Poisson distribution, the log-likelihood function is given by:

$$ L\left(\varTheta |{y}_{t_j}\right)=\sum \limits_{j=1}^n\left[{y}_{t_j}\ \mathit{\log}\left(f\left({t}_j;\varTheta \right)\right)-f\left({t}_j;\varTheta \right)\right] $$

and the Poisson-MLE estimate is expressed as

$$ \hat{\varTheta}= argmax{\sum}_{j=1}^n\left[{y}_{t_j}\ \mathit{\log}\left(f\left({t}_j;\varTheta \right)\right)-f\left({t}_j;\varTheta \right)\right]. $$

In Matlab, we can use the *fmincon* function to set the optimization problem.

To quantify parameter uncertainty, we follow a parametric bootstrapping approach which allows the computation of standard errors and related statistics in the absence of closed-form formulas [19]. As previously described in ref. [34], we generate *B* replicates from the best-fit model $f\left(t,\hat{\varTheta}\right)$ by assuming an error structure in the data (e.g., Poisson) in order to quantify the uncertainty of the parameter estimates and construct confidence intervals. Specifically, using the best-fit model $f\left(t,\hat{\varTheta}\right)$, we generate B-times replicated simulated datasets, where the observation at time *t*_*j*_ is sampled from the Poisson distribution with mean $f\left({t}_j,\hat{\varTheta}\right)$. Next, we refit the model to each of the B simulated datasets to re-estimate parameters for each of the B-simulated realizations. The new parameter estimates for each realization are denoted by ${\hat{\Theta}}_b$ where *b* = 1, 2, …, *B*. Using the sets of re-estimated parameters $\left({\hat{\Theta}}_b\right),$ it is possible to characterize the empirical distribution of each estimate, calculate the variance, and construct confidence intervals for each parameter. Moreover, the resulting uncertainty around the model fit can similarly be obtained from $f\left(t,{\hat{\Theta}}_1\right),$ $f\left(t,{\hat{\Theta}}_2\right),\dots, f\left(t,{\hat{\Theta}}_B\right)$. It is worth noting that a Poisson error structure is the most common for modeling count data where the mean of the distribution equals the variance. In situations where the time series data show over-dispersion, a negative binomial distribution can be employed instead [34]. This parameter estimation method has been shown to perform well with simulated and real epidemic data [30, 34, 36].

### Model-based forecasts with quantified uncertainty

Forecasting from a given model $f\left(t,\hat{\Theta}\right),h$ units of time ahead is given by: $f\left(t+h,\hat{\Theta}\right)$. The uncertainty of the forecasted value can be obtained using the previously described parametric bootstrap method. Let

$$ f\left(t+h,{\hat{\Theta}}_1\right),f\left(t+h,{\hat{\Theta}}_2\right),\dots, f\left(t+h,{\hat{\Theta}}_B\right) $$

denote the forecasted value of the current state of the system propagated by a horizon of h time units, where ${\hat{\Theta}}_b$ denotes the estimation of parameter set Θ from the b_th_ bootstrap sample. We can calculate the bootstrap variance of the estimates to measure the uncertainty of the forecasts, and use the 2.5 and 97.5% percentiles to construct the 95% prediction intervals (PI).

#### Constructing ensemble models

Ensemble approaches aim to combine the strength of multiple models rather than selecting the most promising model and discarding all of the other plausible models which may help enhance predictive performance by contributing important information about the phenomenon under study. Here we introduce two ensemble methods based on different parametric bootstrapping to assess the uncertainty of the ensemble models from a set of dynamic models using differential equations. These ensemble methods differ in the way the variance is evaluated for generating the prediction intervals of the forecasts. Specifically, Ensemble Method 1 is based on the weighted combination of the individual models whereas Ensemble method 2 randomly selects the *i*-th model with probability *w*_*i*_ for each time point of the trajectory of each bootstrap replicate. Below we provide a detailed description of these ensemble methods.

#### Ensemble method 1

Suppose we have *I* models under consideration. Given the training data, let ${\hat{\varTheta}}_i$ denote the set of estimated parameters and ${f}_i\left(t,{\hat{\varTheta}}_i\right)$ denote the estimated mean incident curve, for the *i*-th model. Based on the quality of the model fit measured by the MSE or criteria such as AIC, we compute the weight *w*_*i*_ for the *i*-th model, *i* = 1, …, *I*, where ∑*w*_*i*_ = 1. For instance, if we use the mean squared error (MSE) to assess the quality of the model fit then the weight for each individual model is given by:

$$ {w}_i=\frac{\frac{1}{MSE_i}}{\frac{1}{MSE_1}+\frac{1}{MSE_2}+\dots +\frac{1}{MSE_I}}\ \mathrm{for}\ \mathrm{all}\ i=1,2,\dots, I,\mathrm{where}\ {MSE}_i=\frac{1}{n}{\sum}_{j=1}^n{\left({f}_i\left({t}_j,{\hat{\varTheta}}_i\right)-{y}_{t_j}\right)}^2. $$

Hence, the estimated mean incidence curve from the ensemble model is:

$$ {f}_{ens}(t)=\sum \limits_{i=1}^I{w}_i{f}_i\left(t,{\hat{\Theta}}_i\right) $$

Assuming that the observed incidence series have a Poisson (or negative binomial) distribution

with mean *f*_*ens*_(*t*), we can construct the 95% CI or PI for the incidence at time *t* using the parametric bootstrap method for the ensemble method. Specifically, suppose the training sample size is n with time points *t*_1_, …, *t*_*n*_. To generate a Bootstrap sample, we generate a random variable *y*_*i*_ from Poisson distribution with mean *f*_*ens*_(*t*_*j*_):

$$ {y}_j\sim Poisson\left({f}_{ens}\left({t}_j\right)\right)\mathrm{for}\ j=1,\dots, \mathrm{n}. $$

**Fig. 1:**
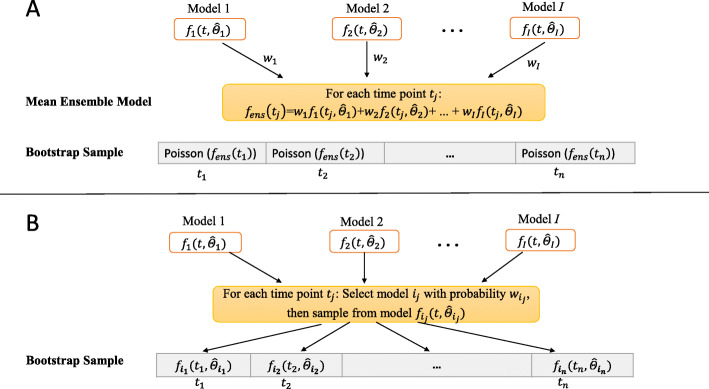
Schematic diagrams illustrate the construction of the Bootstrap samples using Ensemble Method 1 (**a**) and Ensemble Method 2 (**b**). Suppose we have *I* models under consideration. Given the training data, let ${\hat{\varTheta}}_i$ denote the set of estimated parameters and ${f}_i\left(t,{\hat{\varTheta}}_i\right)$ denote the estimated mean incident curve, for the *i*-th model. Based on the quality of the model fit measured by the MSE or criteria such as AIC, we compute the weight *w*_*i*_ for the *i*-th model, *i* = 1, ..., *I*, where ∑*w*_*i*_ = 1. For Method 1, we generate a random variable *y*_*i*_ from Poisson distribution with mean ${f}_{ens}\left({t}_j\right)=\sum \limits_{i=1}^I{w}_i{f}_i\left(t,{\hat{\Theta}}_i\right)$ to generate a bootstrap sample. In contrast, to generate the Bootstrap samples based on Method 2, we assume that at each time point the epidemic follows the *i*-th model with probability *w*_*i*_

Then { *y*_1_, …, *y*_*n*_ } is a bootstrap sample, from which we can re-fit each of the *I* models, calculate weights, and get the estimate and generate the ensemble model’s forecast. Doing this *B* times, we can construct the 95% CI or prediction interval using the 2.5 and 97.5% quantiles. This method assumes that the whole population consists of *I* sub-populations, and the *i*-th subpopulation follows model *i*. The total incidence is the sum of incidences from *I* sub-populations with the *i*-th subpopulation accounting for *w*_*i*_ of the whole population. For this method the mean and variance of the ensemble are both equal to *f*_*ens*_(*t*_*j*_). Figure 1a illustrates the construction of the Bootstrap sample according to Ensemble Method 1.

#### Ensemble method 2

This method differs from Ensemble Method 1 in the way the Bootstrap samples are generated for the fitted ensemble model. Specifically, to generate the Bootstrap samples, we assume that at each time point the epidemic follows the *i*-th model with probability *w*_*i*_. Then we can generate the *b*-th bootstrap sample as follows. At each time point *t*_*j*_, *j* = 1, …, n,

1. Choose model: Generate a random variable *i* from the set {1, …, *I*} with corresponding probability set { *w*_1_, …, *w*_*I*_ }. Suppose that the *i*-th model is chosen.
2. Given that the *i*-th model is chosen, generate a random variable *y*_*i*_ from the Poisson distribution with mean ${f}_i\left({t}_{j,}{\hat{\varTheta}}_i\right)$:
  $$ {y}_j\sim Poisson\left({f}_i\left({t}_{j,}{\hat{\varTheta}}_i\right)\right) $$

Then { *y*_1_, …, *y*_*n*_ } forms a bootstrap sample. The marginal mean of *y*_*j*_ is ${f}_{ens}\left({t}_j\right)={\sum}_{i=1}^I{w}_i{f}_i\left({t}_j,{\hat{\varTheta}}_i\right)$ and the marginal variance is

$$ {f}_{ens}\left({t}_j\right)+\sum \limits_{i=1}^I{w}_i{f}_i^2\left({t}_{j,}{\hat{\varTheta}}_i\right)-{f}_{ens}^2\left({t}_j\right)=\sum \limits_{i=1}^I{w}_i{f}_i\left(t,{\hat{\varTheta}}_i\right)+\sum \limits_{i=1}^I{w}_i{f}_i^2\left({t}_{j,}{\hat{\varTheta}}_i\right)-{\left\{\sum \limits_{i=1}^I{w}_i{f}_i\left(t,{\hat{\varTheta}}_i\right)\right\}}^2 $$

which is larger than *f*_*ens*_(*t*_*j*_), the variance of the ensemble model derived from the Ensemble Method 1. Figure 1b illustrates the construction of the Bootstrap sample using Ensemble Method 2. In summary, Ensemble Method 1 takes the occurrence of each model as deterministic with the proportion of new cases taken from each model at each time point specified as *w*_*i*_. Thus, the total number of new cases is the weighted average of all models. In contrast, Ensemble Method 2 takes the occurrence of each model as random at each time point, with the probability of the occurrence of the *i*-th model given by *w*_*i*_. Hence the expected value is the weighted average of all models, and the weights correspond to the probabilities for each model. However, the randomness in the occurrence of the models across time points introduces additional variation in the ensemble estimates, leading to higher variance than the first ensemble method.

#### Models for short-term forecasting the trajectory of epidemics

To illustrate our ensemble methodology, we employ simple dynamic growth models which have been previously used in various disease forecasting studies (e.g. [4, 39–42]). Specifically, we conducted a comparative study to assess the forecasting performance of the ensemble methods that combine three dynamic growth models based on simulated and real epidemic datasets. Below we describe the single models that we use to construct the ensemble model, where *C*(*t*) denotes the cumulative case count at time *t*.

#### Generalized logistic model (GLM)

The Generalized Logistic model (GLM) has 3 parameters and is given by:

$$ \frac{dC(t)}{dt}={C}^{\prime }(t)=r{C}^p(t)\left(1-\frac{C(t)}{K_0}\right) $$

The scaling of growth parameter, *p*, is also used in the GGM to model a range of early epidemic growth profiles ranging from constant incidence (*p* = 0), polynomial (0 < *p* < 1) and exponential growth dynamics (*p* = 1). The remaining model parameters are as follows: *r* is the growth rate, and *K*_0_ *K* is the final epidemic size. For this model, we estimate Θ = (*r*, *p*, *K*_0_) where *f*(*t*, Θ) = *C*^′^(*t*) and fix the initial number of cases *C*(0) according to the first observation in the data. The GLM model has been employed to generate short-term forecasts of Zika, Ebola, and COVID-19 epidemics [8, 9, 39, 43]. In particular, forecasts from the GLM model based on the initial growth phase of an epidemic tend to under predict disease incidence before the inflection point has occurred.

#### Richards model (RIC)

The well-known Richards model is an extension of the simple logistic growth model and relies on 3 parameters. It extends the simple logistic growth model by incorporating a scaling parameter, *a*, that measures the deviation from the symmetric simple logistic growth curve [34, 44, 45]. The Richards model is given by the differential equation:

$$ \frac{dC(t)}{dt}= rC(t)\left[1-{\left(\frac{C(t)}{K_0}\right)}^a\right] $$

where *r* is the growth rate, *a* is a scaling parameter and *K*_0_ is the final epidemic size. The Richards model has been employed to generate short-term forecasts of SARS, Zika, Ebola, and COVID-19 epidemics [8, 9, 39, 43, 46].

#### Gompertz model (GOM)

The 2-parameter Gompertz model is given by:

$$ \frac{dC(t)}{dt}={C}^{\prime }(t)= rC(t){e}^{- bt} $$

Where *r* is the growth rate and *b* > 0 describes the exponential decline of the growth rate. For this model, we estimate Θ = (*r*, *b*) where *f*(*t*, Θ) = *C*^′^(*t*) and fix the initial number of cases *C*(0) according to the first observation in the data. The GOM model has been employed to generate short-term forecasts of Zika and COVID-19 epidemics [40, 47, 48].

#### Forecasting strategy and performance metrics

Using the GLM, RIC, GOM, and two ensemble methods described above, we conducted sequential *h*-time units ahead forecasts where *h* ranged from 1 to 20 days for daily time series data, and from 1 to 4 weeks for the weekly outbreak scenarios of the *Ebola Forecasting Challenge*. Each of these models were sequentially re-calibrated starting from the first data point using the most up-to-date incidence curve. That is, the calibration period for each sequential forecast included one additional data point than the previous forecast.

To assess forecasting performance, we used four performance metrics: the mean absolute error (MAE), the mean squared error (MSE), the coverage of the 95% prediction intervals, and the mean interval score (MIS) [49]. The *mean absolute error* (MAE) is given by:

$$ \mathrm{MAE}=\frac{1}{N}\sum \limits_{i=1}^N\left|f\left({t}_i,\hat{\varTheta}\right)-{y}_{t_i}\right| $$

Here ${y}_{t_i}$ is the time series of incident cases of the h-time units ahead forecasts where *t*_*i*_ are the time points of the time series data [50]. Similarly, the *mean squared error* (MSE) is given by:

$$ \mathrm{MSE}=\frac{1}{N}\sum \limits_{i=1}^N{\left(f\left({t}_i,\hat{\varTheta}\right)-{y}_{t_i}\right)}^2 $$

We also employed two metrics that account for prediction uncertainty: The *coverage rate of the 95% prediction interval*, e.g., the proportion of the observations that fall within the 95% prediction interval as well as the *mean interval score* (MIS) [49, 51] which is a proper score that evaluates the width of the 95% prediction interval as well as coverage which is given by:

$$ \mathrm{MIS}=\frac{1}{h}\sum \limits_{i=1}^h\left[\left({U}_{t_i}-{L}_{t_i}\right)+\frac{2}{0.05}\left({L}_{t_i}-{y}_{t_i}\right)I\left\{{y}_{t_i}<{L}_{t_i}\right\}+\frac{2}{0.05}\left({y}_{t_i}-{U}_{t_i}\right)I\left\{{y}_{t_i}>{U}_{t_i}\right\}\right] $$

where *L*_*t*_ and *U*_*t*_ are the lower and upper bounds of the 95% prediction interval and *Ι*{} is an indicator function. Thus, this metric rewards for narrow 95% prediction intervals and penalizes at the points where the observations are outside the bounds specified by the 95% prediction interval where the width of the prediction interval adds up to the penalty (if any) [49].

The mean interval score (MIS) and the coverage of the 95% prediction intervals take into account the uncertainty of the predictions whereas the mean absolute error (MAE) and mean squared error (MSE) only assess the closeness of the mean trajectory of the epidemic to the observations [13]. These performance metrics have been adopted in the international *M4 forecasting competition* [52] and more recent studies that systematically compare forecasting performance in the context of the 2018–19 Ebola epidemic in DRC [7, 41] and the COVID-19 pandemic [8].

#### Testing and verification of ensemble methods using synthetic data

Before applying the new ensemble methods to real epidemic contexts, it is important to demonstrate the functionality of the ensemble methodology through simulation studies. Specifically, we constructed ensemble models using three individual models (GLM, RIC, GOM) based on the quality of the model fit to the data. For this purpose, we considered two sources of synthetic data as follows:

- Simulated daily incidence curve from the Gompertz model (GOM), which is one of the three models used to construct the ensemble model.
- Synthetic data generated using a stochastic SEIR model that incorporates a time-dependent transmission rate to model more temporal variability in the incidence curve. We assessed the forecasting performance (1-day to 20-day ahead forecasts) achieved by each of three individual models (GLM, RIC, GOM) as well as the two ensemble models. In particular, we are interested in assessing how well the ensemble methods perform relative to the individual models. Below we provide a detailed description of the synthetic data generation process.

##### Synthetic data generated from the Gompertz model

**Fig. 2:**
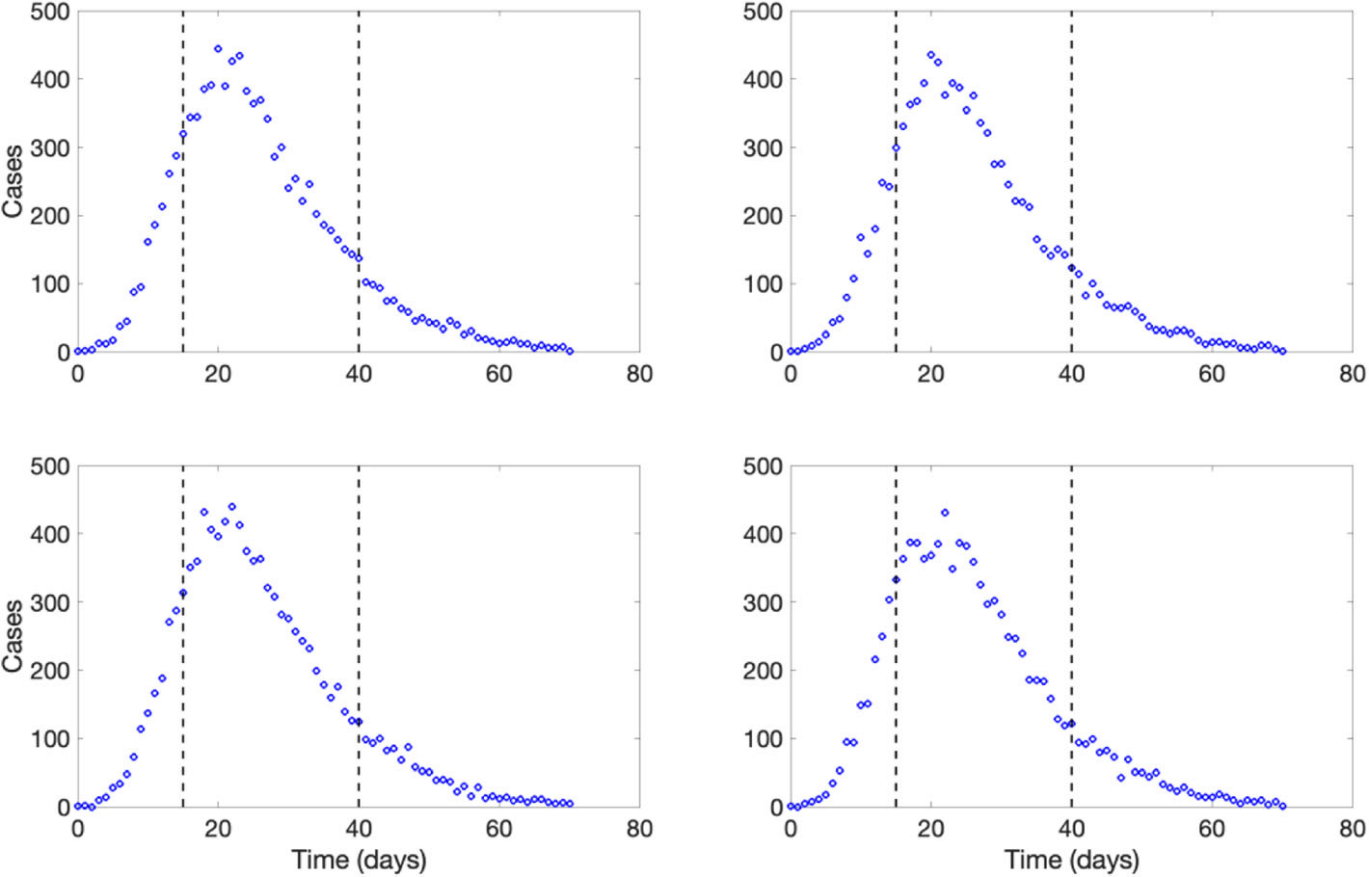
Synthetic datasets for testing and demonstrating the functionality of the ensemble approaches. We simulated incidence curves from the 2-parameter Gompertz model (the “true model”) with added Poisson error structure noise (blue circles). We set parameters *r* = 0.4, *b* = 0.1086 and *K* = 10,000. The initial condition was set at *C*(0) = 1. The dashed vertical lines indicate the start and end days of the daily 20-day ahead forecasts

We simulated incidence curves from the 2-parameter Gompertz model (the “true model”) with Poisson noise (Fig. 2). Then we used the simulated epidemic curves to assess the forecasting performance by each of three individual models (GLM, RIC, GOM), a set that includes the “true model”, as well as the two ensemble models in 1-day to 20-day ahead forecasts. We expect the “true model” (GOM) to outperform all of the individual models as well the ensemble models. We also expect that the ensemble models will outperform, on average, the individual models except for the “true model” (GOM). To generate synthetic data, we selected the GOM parameters such that the total number of cases by the end of the epidemic is 10,000 [53]. Thus,

$$ r=1-\frac{C(0)}{10000}\ \mathrm{and}\ b=\frac{r}{\ln \left(\frac{10000}{C(0)}\right)}\ \mathrm{where}\ C(0)=1. $$

##### Synthetic data from a stochastic SEIR model with time-dependent transmission rate

**Fig. 3:**
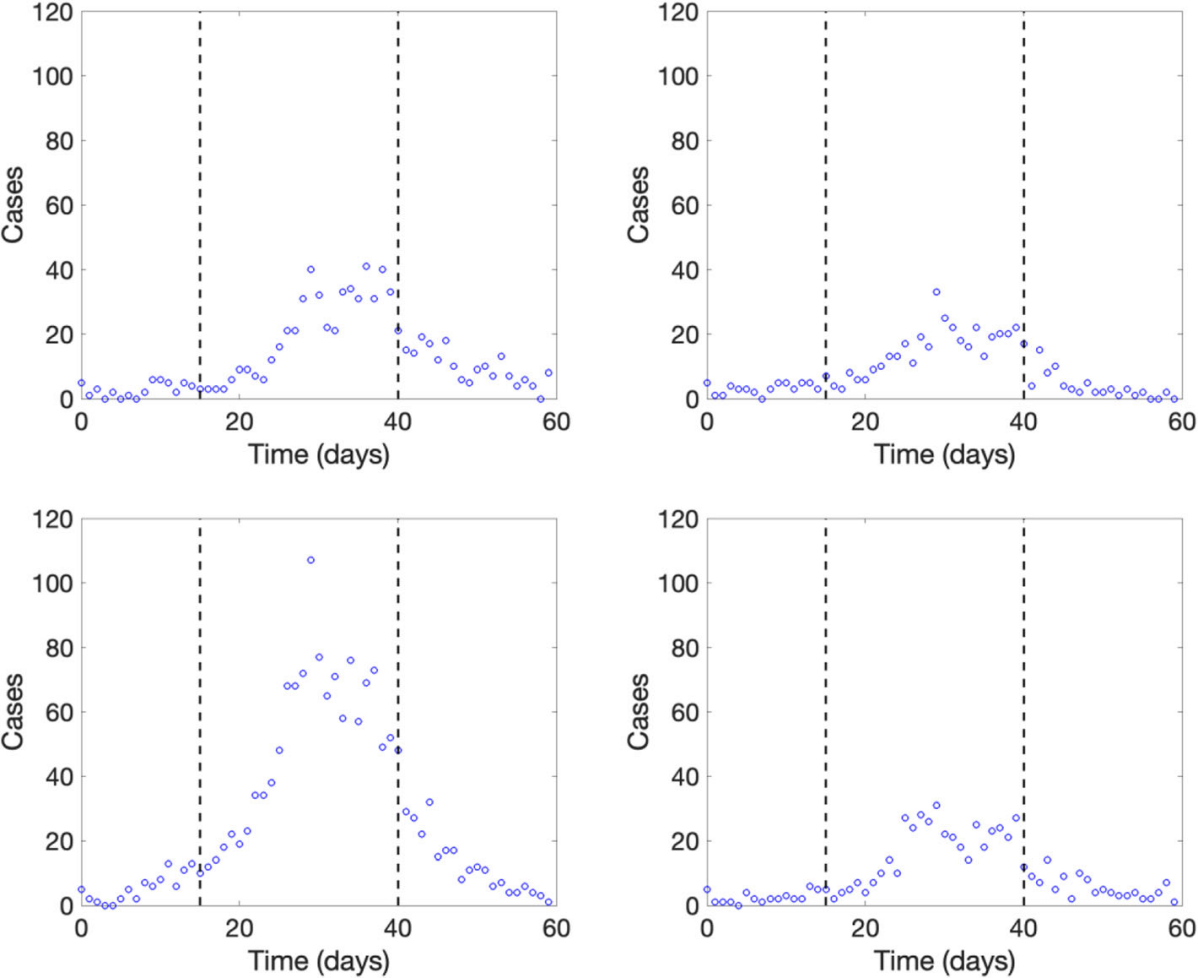
Synthetic datasets derived from a stochastic homogenous-mixing SEIR transmission model with a population size of 100,000 and time-dependent transmission rate such that the resulting incidence curves are not well-captured by any of the individual models considered in the ensemble model (GLM, RIC, GOM). These simulations have a constant reproduction number of 2.0 from day 0 to day 20, then the reproduction number declines from 2.0 to 1.0 on epidemic day 30 and then finally the reproduction number drops from 1.0 to 0.5 on epidemic day 40. The simulations start with 5 infected individuals. The dashed vertical lines indicate the start and end days of the daily 20-day ahead forecasts

We generated simulated data using an SEIR transmission model with time-dependent transmission rate *β*(*t*), a model that is not included in the ensemble models. Specifically, we generated stochastic realizations from a homogenous-mixing SEIR model with a population size of 100,000 and time-dependent transmission rate such that the resulting incidence curves display a brief leveling off before a decay phase, a pattern that is not well-captured by any of the individual models employed to construct the ensemble model (GLM, RIC, GOM). More specifically, we generated stochastic simulations with a constant reproduction number of 2.0 from day 0 to day 20, then the reproduction number declines to near endemicity from R = 2.0 to R = 1.0 on epidemic day 30. Finally, the reproduction number drops from 1.0 to 0.5 on epidemic day 40. Thus, these epidemic curves exhibit an exponential growth period from day 0 to day 20, then a brief steady incidence trend from day 30 to day 40 before the number of new cases declines towards zero (Fig. 3).

#### The Ebola forecasting challenge

We also assessed the forecasting performance of the ensemble and individual models using four synthetic epidemic trajectories (scenarios) from the *Ebola Forecasting Challenge* [4], an effort that was inspired by the 2014–2015 West African Ebola outbreak and generated based on a detailed individual-based transmission model for Liberia [54]. These synthetic epidemics have different levels of data quality and quantity based on different epidemiological conditions, behavioral changes, and intervention measures (Figure S1). For Scenarios 1–3, interventions bring the epidemic under control while Scenario 4 represents an uncontrolled outbreak that included a temporary downturn in case incidence [4]. All of the models were calibrated for each scenario starting from week 0. For each of the four scenarios, we generated weekly forecasts based on the first and last forecasting periods defined in the *Ebola Forecasting Challenge* [4]. For instance, for Scenario 1, we generated a total of 23 short-term forecasts from day 20 until day 42 (Figure S1).

#### Real outbreak data

We applied our new ensemble modeling methods to generate short-term forecasts for eight real epidemics namely Zika in Antioquia, Colombia, the 1918 influenza pandemic in San Francisco, the 2009 A/H1N1 influenza pandemic in Manitoba, Canada, severe acute respiratory syndrome (SARS) in Singapore, plague in Madagascar, and COVID-19 epidemics in the provinces of Guangdong, Henan and Hunan [55].

### Zika in Antioquia, Colombia

**Fig. 4:**
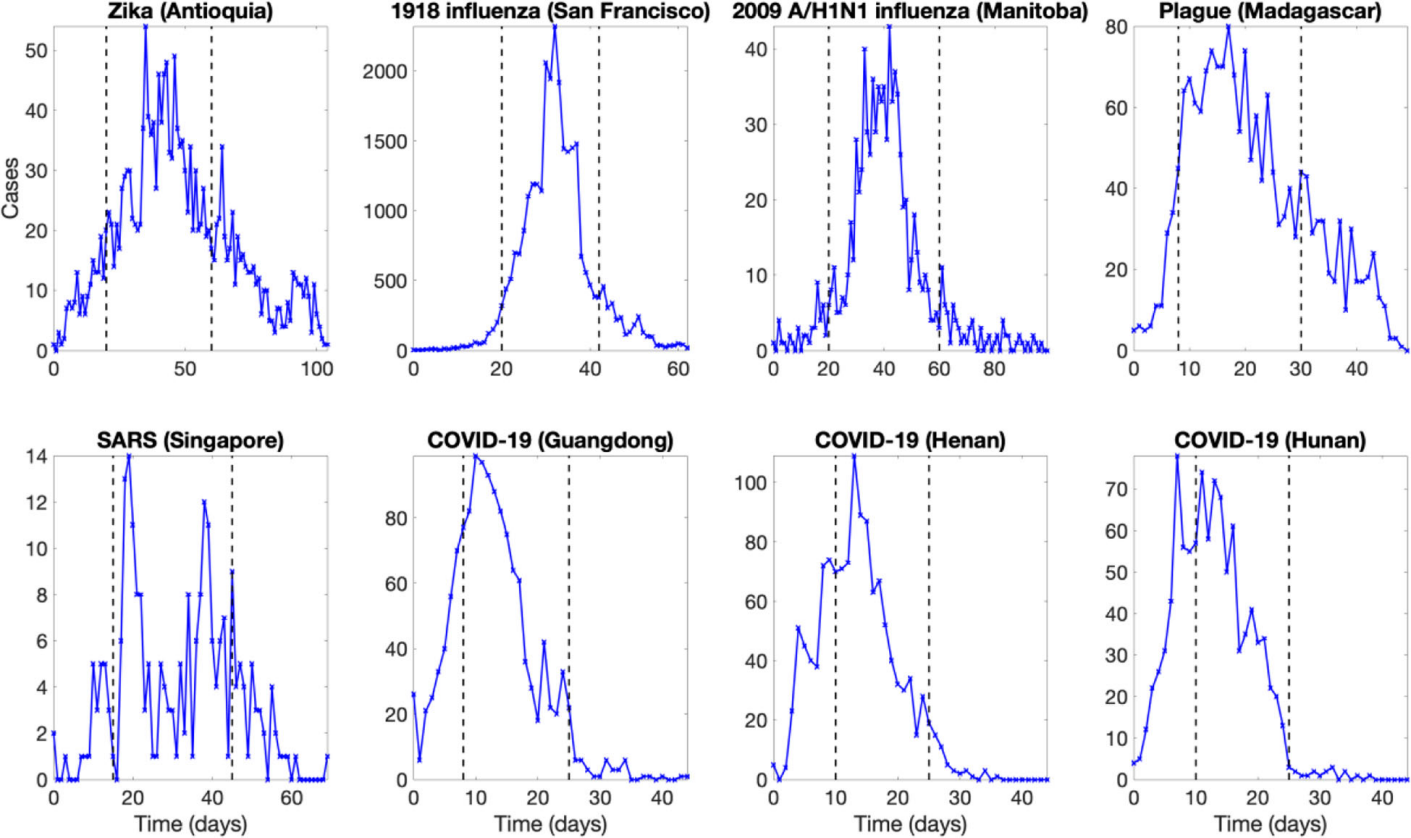
Epidemic trajectories for eight real epidemics namely Zika in Antioquia, Colombia, the 1918 influenza pandemic in San Francisco, the 2009 A/H1N1 influenza pandemic in Manitoba, Canada, Severe Acute Respiratory Syndrome (SARS) in Singapore, plague in Madagascar, and COVID-19 epidemics in the provinces of Guangdong, Anhui, and Hunan. The dashed vertical lines indicate the start and end days of the daily 20-day ahead forecasts

We analyzed daily counts of suspected Zika cases by date of symptoms onset of the 2016 outbreak in Antioquia, Colombia [39]. Antioquia is the second largest department in the central northwestern part of Colombia (with a population size of 6.3 million people). The epidemic wave peaked 36 days into the outbreak. For each model, we generated daily short-term forecasts from day 20 until day 60 (Fig. 4).

### The 1918 influenza pandemic in San Francisco, California

We analyzed the daily epidemic curve of reported cases during the fall wave of the 1918 influenza pandemic in San Francisco, California [56]. A total of 28 310 cases including 1908 deaths were attributed to the fall epidemic wave comprising 63 epidemic days with the first case reported on 23 September 1918. For each model, we generated daily short-term forecasts from day 20 until day 42 (Fig. 4).

### 2009 A/H1N1 influenza in Manitoba, Canada

Daily number of laboratory-confirmed cases of H1N1 influenza infection were obtained from influenza databases of Manitoba Health for both waves of the 2009 pandemic in spring (total of 891 cases between May 2 and August 5) and fall (total of 1774 cases between October 1, 2009, and January 3, 2010), classified for each of the 11 health regions in the province of Manitoba, Canada. A laboratory-confirmed case was defined as an individual with influenza-like illness or severe respiratory illness who tested positive for pandemic H1N1 influenza A virus by real-time reverse-transcriptase PCR (RT-PCR) or viral culture. The first case of H1N1 infection in Manitoba was identified (tested positive) on May 2, 2009 [57]. For each model, we generated daily short-term forecasts from day 20 until day 60 (Fig. 4).

### Plague outbreak in Madagascar

We analyzed the main epidemic wave of the 2017 plague epidemic in Madagascar which was retrieved from the WHO reports. The epidemic wave consists of weekly confirmed, probable and suspected plague cases during September–November 2017 [58]. For each model, we generated daily forecasts from day 8 to day 30 (Fig. 4).

### SARS outbreak in Singapore

We obtained the daily number of new SARS cases by date of symptom onset of the 2003 SARS outbreak in Singapore [59]. This outbreak involved three major hospitals in Singapore, and the incidence curve exhibited a bimodal shape with two peaks occurring in mid-March and early April (2003), respectively. These two small sub-epidemics largely correspond to outbreaks stemming from different healthcare settings [59]. This epidemic lasted a total of 70 days. For each model, we generated daily short-term forecasts from day 15 until day 45 (Fig. 4).

### COVID-19 outbreaks in Guangdong, Henan and Hunan

We used data from the National Health Commission of China which reports the cumulative cases for provinces, including municipalities, autonomous regions, and special administrative regions [60]. We collected reported case data each day at 12 pm (GMT-5) from the initial date of reporting, 22 January 2020 to 25 April 2020. We focused on the provinces of Guangdong, Anhui, and Hunan, which have exhibited a high burden of COVID-19. For Guangdong Province, we conducted daily forecasts from day 8 to day 25; for Anhui and Hunan Provinces, we conducted forecasts from day 10 to day 25 (Fig. 4).

## Results

**Fig. 5:**
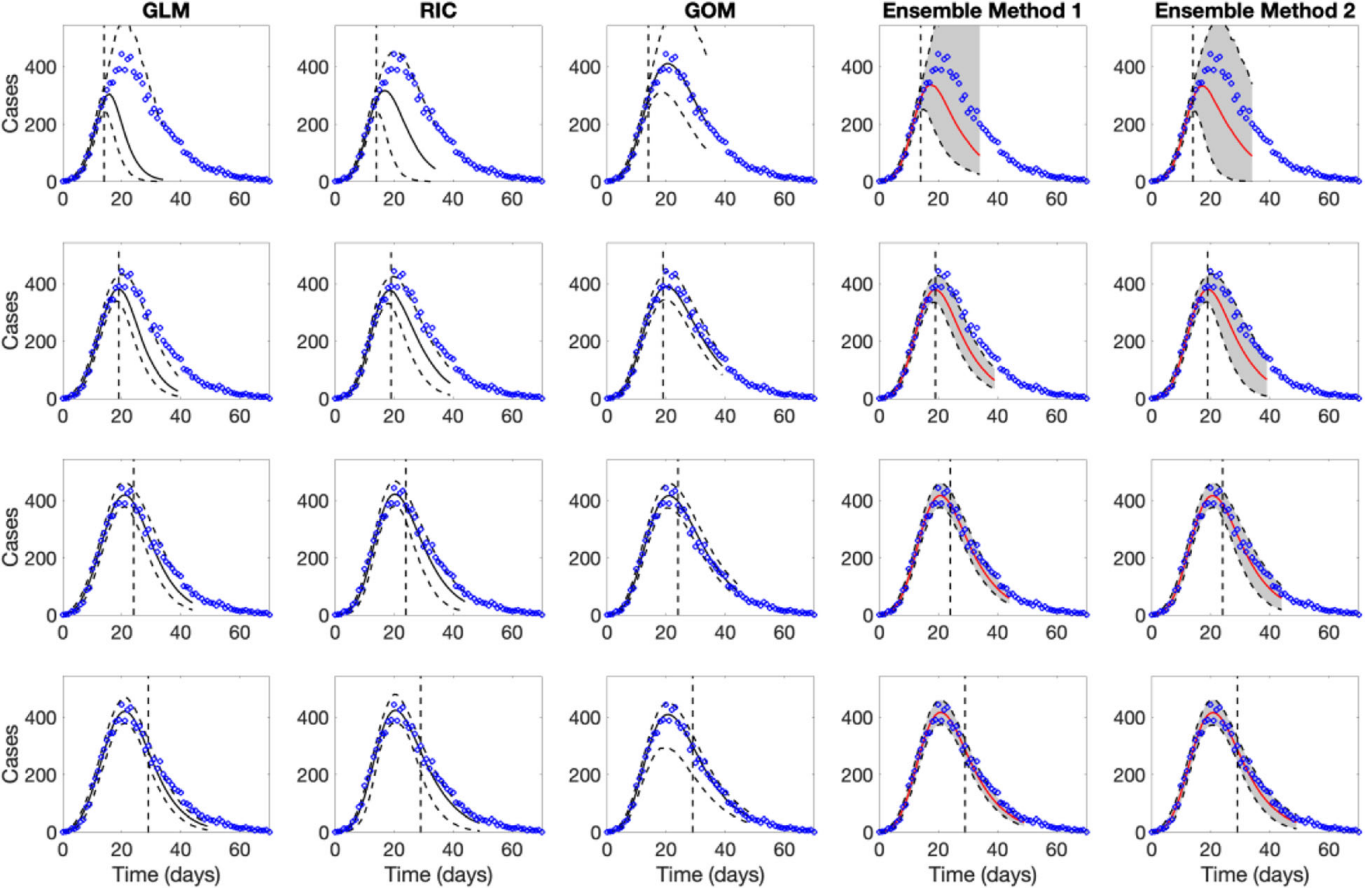
Representative sequential 20-day ahead forecasts (top to bottom panels) obtained from individual models (GLM, RIC, GOM) and two ensemble methods applied to synthetic data derived from the GOM model. Blue circles correspond to the data points. The mean fit (solid line) and 95% prediction interval (dashed lines) are also shown. The gray shaded areas help highlight differences in the 95% prediction intervals associated with the ensemble methods. The vertical line separates the calibration period (left) from the forecasting period (right)

**Fig. 6:**
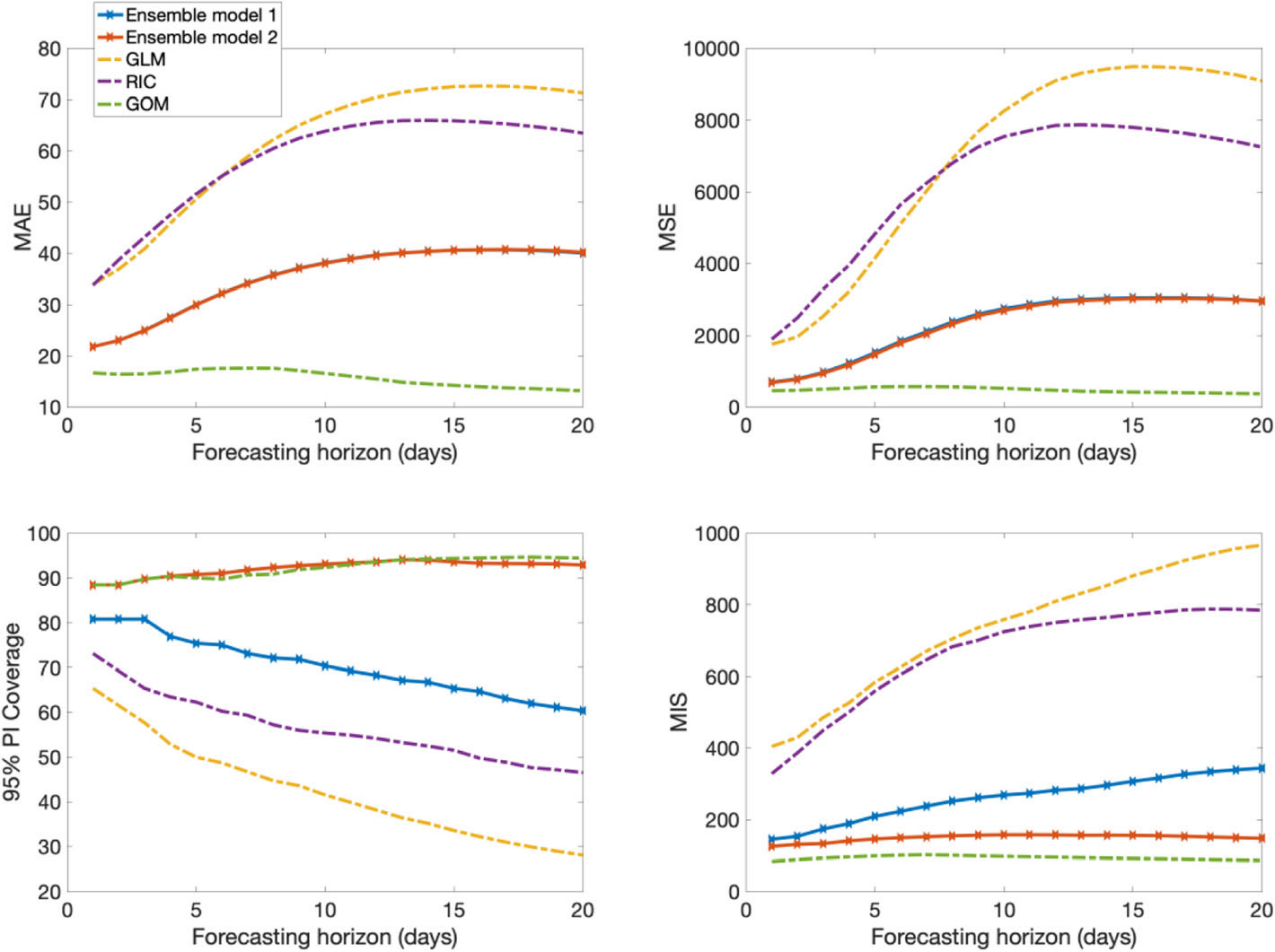
Mean performance of the individual and ensemble models in 1–20 day ahead forecasts from the synthetic data derived from the Gompertz model. As expected, we found that the “true model” (GOM) outperformed all other models based on four performance metrics although it achieved a similar coverage rate of the 95% PI to that of the Ensemble Method 2, which was close to 0.95. While the performance of the ensemble methods was not different in terms of the MAE and MSE, Ensemble Method 2 achieved significantly better coverage rate of the 95% PI and lower MIS compared to the Ensemble Method 1

Using synthetic incidence curves simulated from the Gompertz model (Fig. 2), we demonstrated the functionality of the ensemble methods in 20-day ahead forecasts relative to three individual models (GLM, RIC, GOM), a set that includes the “true model”. A set of representative sequential forecasts from all models are shown in Fig. 5. As expected, we found that the “true model” (GOM) outperformed all other models based on all four performance metrics although it achieved a similar coverage rate of the 95% PI to that of the Ensemble Method 2, which was close to 0.95, indicating well-calibrated models (Fig. 6). While the ensemble methods performed similarly in terms of the MAE and MSE, Ensemble Method 2 achieved significantly better coverage rate of the 95% PI and lower MIS compared to the Ensemble Method 1 (Fig. 6). For instance, in 20-day ahead forecasts, the 95% PI of the Ensemble Method 2 covered 92.3% of the data, on average, whereas the Ensemble Method 1 only covered 53.3% of the data. Moreover, the Ensemble Method 2 achieved a lower average MIS (169.1) compared to the ensemble method 1 (371.1). It is also worth pointing out that the coverage rate and MIS achieved by the Ensemble Method 2 were stable across forecasting horizons.

We also assessed the performance of the Ensemble Methods relative to individual models using simulated data from a stochastic SEIR model with time-dependent changes in transmission rate (Fig. 3). A set of representative sequential forecasts from all models are shown in Figure S2. We found that the Ensemble Method 2 outperformed all other models including Ensemble Method 1 based on the coverage rate of the 95% PI and the MIS (Figure S3). Although the RIC model achieved better MAE and MSE compared to the other models, Ensemble Method 2 outperformed the other models including the Ensemble Method 1 based on the performance metrics that account for predictive uncertainty. Furthermore, the coverage rate and MIS were more stable across forecasting horizons for the Ensemble Method 2 compared to the Ensemble Method 1. For instance, for 10- and 20-day ahead forecasts, the 95% PI of the ensemble method 2 covered 91 and 95.2% of the data, respectively. In contrast, the 95% PI of the ensemble method 1 covered 79.5 and 61.9% of the data on average for 10- and 20-day ahead forecasts.

For Scenario 1 of the Ebola challenge, the Ensemble Method 2 achieved consistently better performance across all metrics and forecasting horizons compared to the Ensemble Method 1 and the individual models (Figures S4 and S5). For instance, for 4-week ahead forecasts, the 95% PI of the ensemble method 2 covered 89.2% of the data on average whereas the ensemble method 1 only covered 75.8.3% of the data. Moreover, the ensemble method 2 achieved a lower average MIS (490.2) compared to the ensemble method 1 (615.7). For Scenario 2, the Richards model yields better MIS, but it did not achieve much greater advantage over the Ensemble Method 2 in terms of the coverage rate (Figures S6 and S7). For Scenario 3, GLM and RIC achieved lower MAE, MSE, and better coverage rate. In terms of the MIS, GLM, RIC and Ensemble Method 2 achieved better performance (Figures S8 and S9). Finally, for Scenario 4 characterized by an unmitigated epidemic, the Ensemble Method 2 clearly outperformed all other models including the Ensemble Method 1 (Figures S10 and S11).

**Fig. 7:**
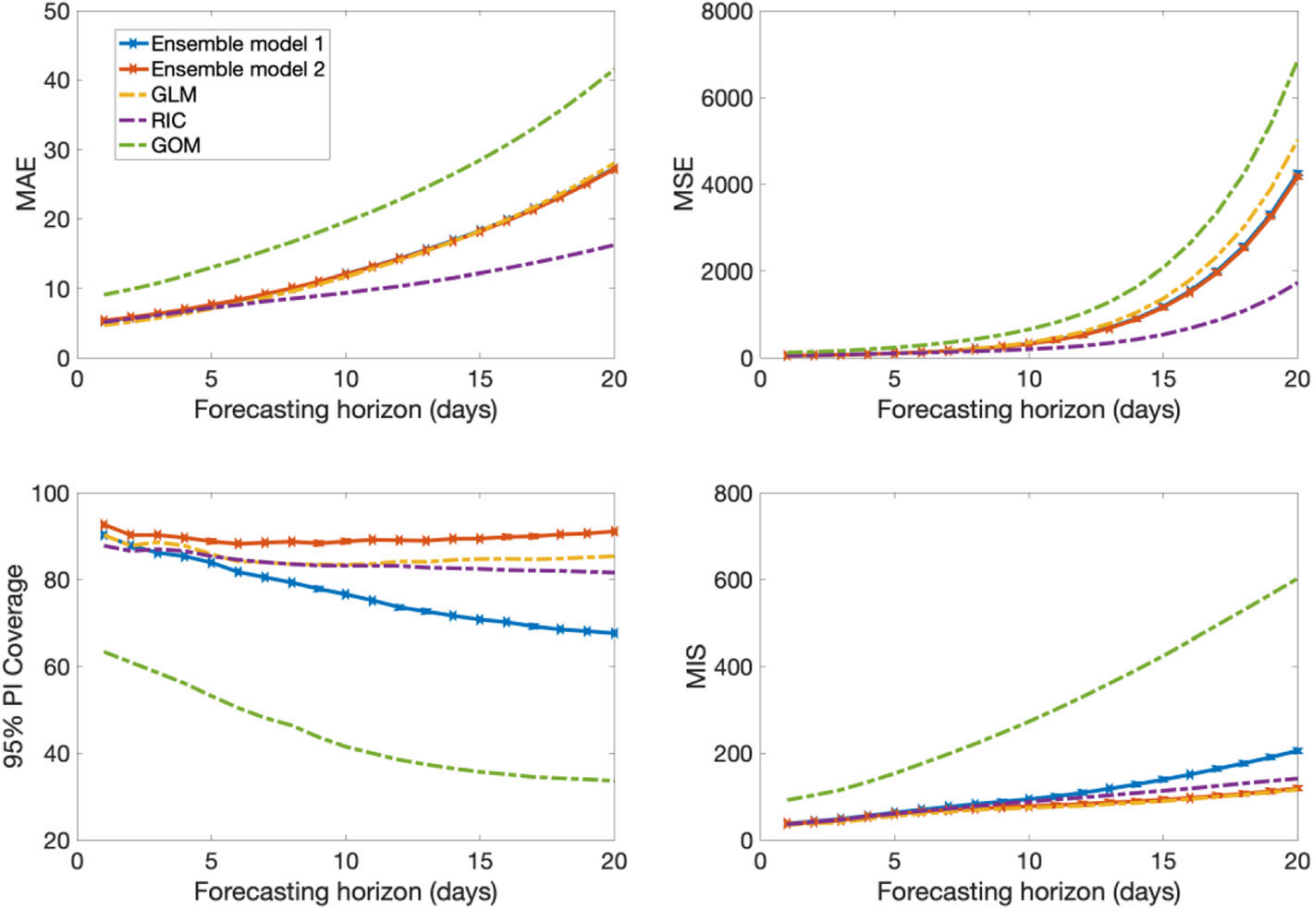
Mean performance of the individual and ensemble models in 1–20 day ahead forecasts for the 2009 A/H1N1 influenza pandemic in Manitoba, Canada. The Ensemble Method 2 outperformed all other models based on the coverage rate of the 95% PI and the MIS albeit predictions were a little away from the actual future values and individual models often attained lower MAE or MSE

**Fig. 8:**
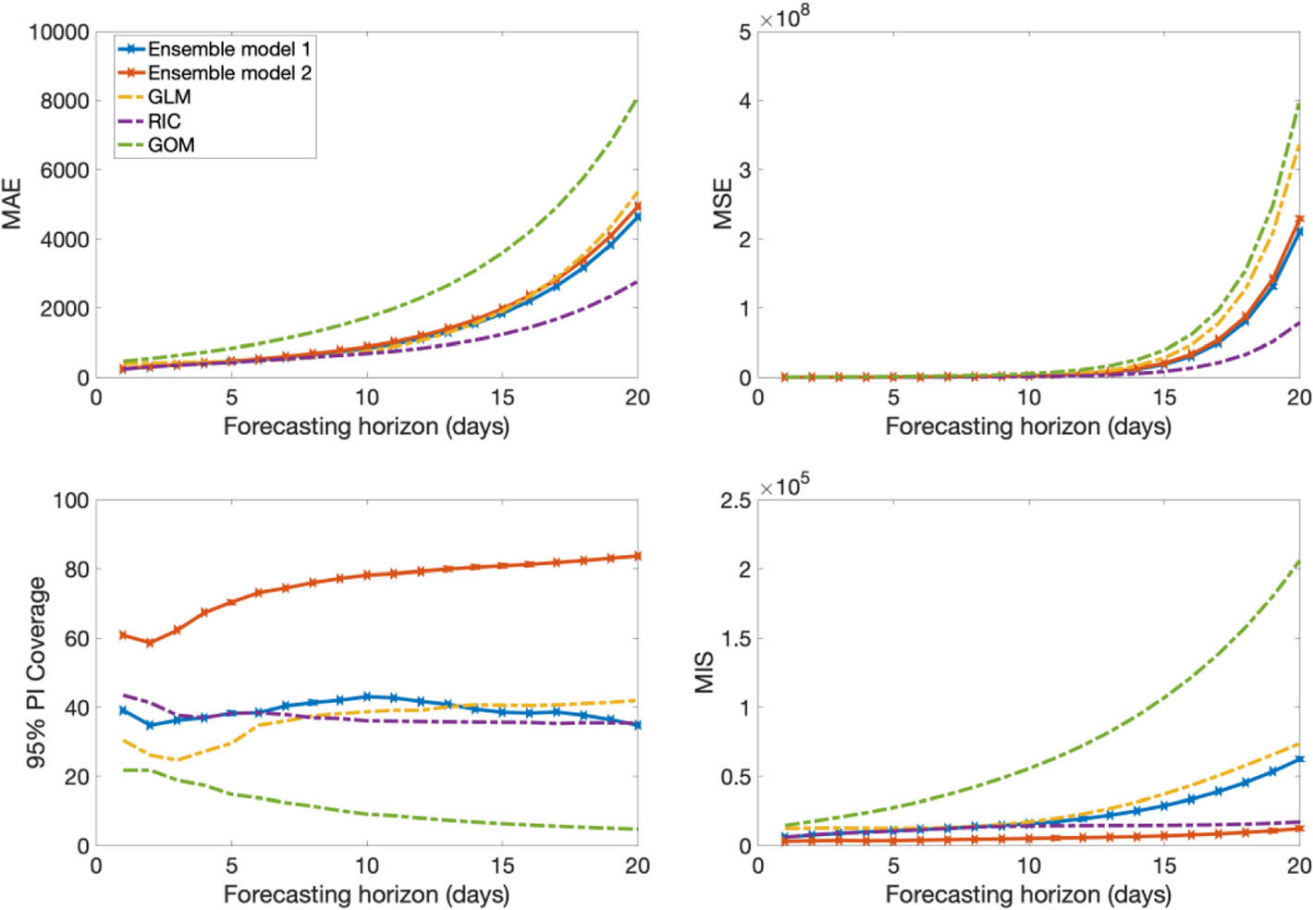
Mean performance of the individual and ensemble models in 1–20 day ahead forecasts for the 1918 influenza pandemic in San Francisco. The Ensemble Method 2 outperformed all other models based on the coverage rate of the 95% PI and the MIS

**Fig. 9:**
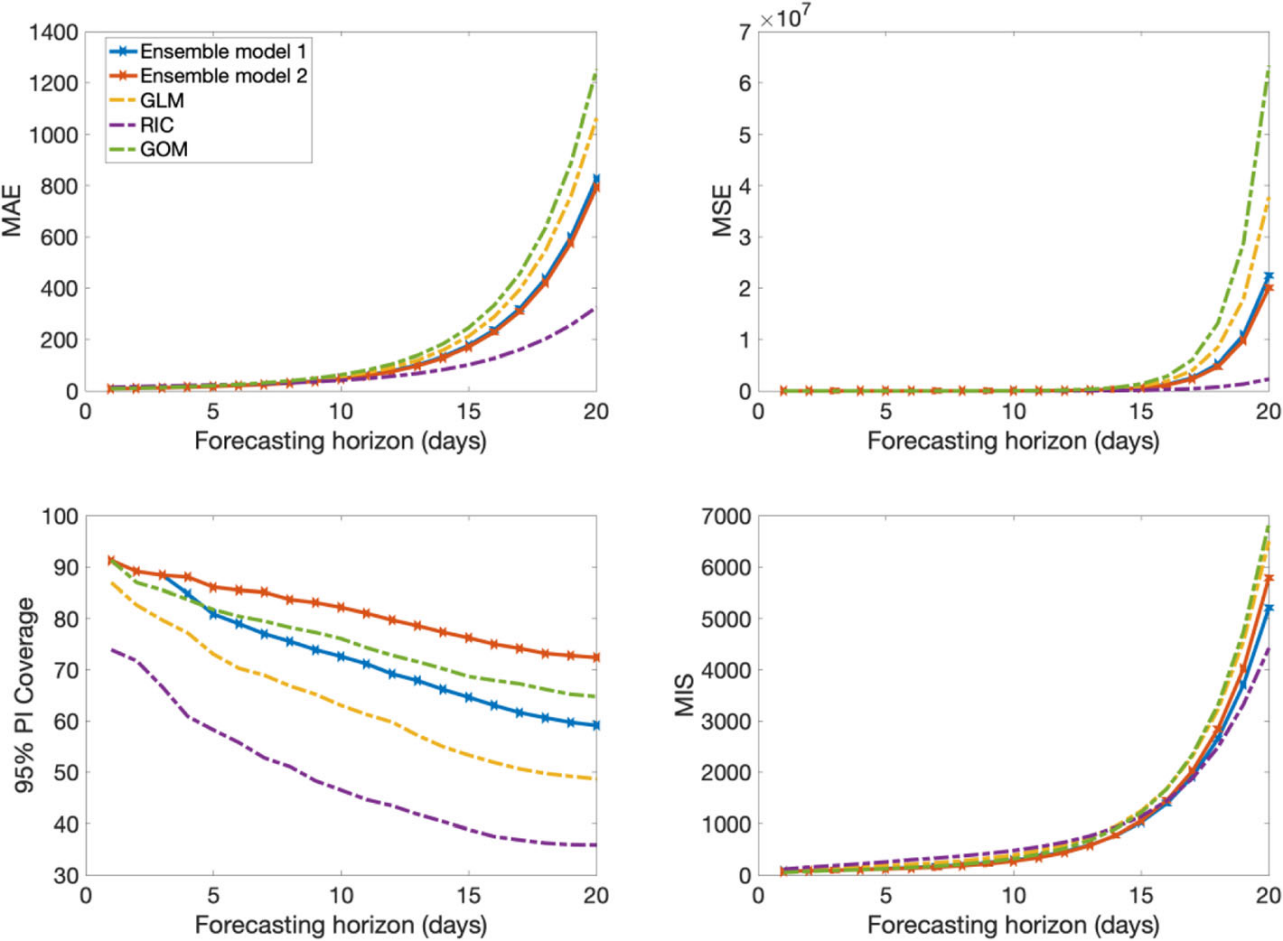
Mean performance of the individual and ensemble models in 1–20 day ahead forecasts for the plague epidemic in Madagascar. The Ensemble Method 2 outperformed all other models based on the coverage rate of the 95% PI and the MIS

**Fig. 10:**
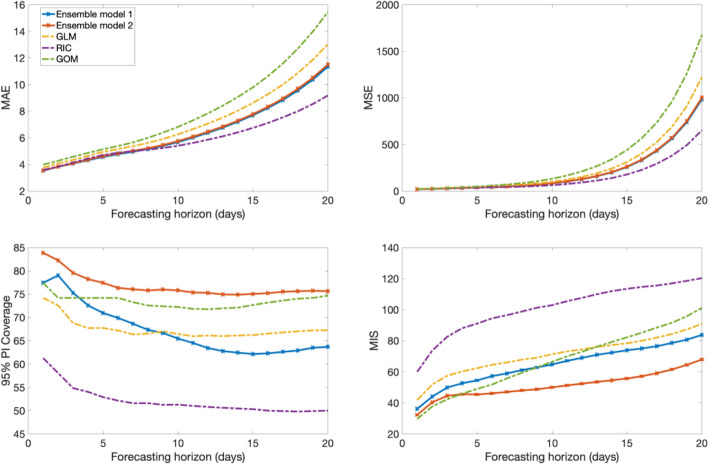
Mean performance of the individual and ensemble models in 1–20 day ahead forecasts for the SARS outbreak in Singapore. The Ensemble Method 2 outperformed all other models based on the coverage rate of the 95% PI and the MIS

**Fig. 11:**
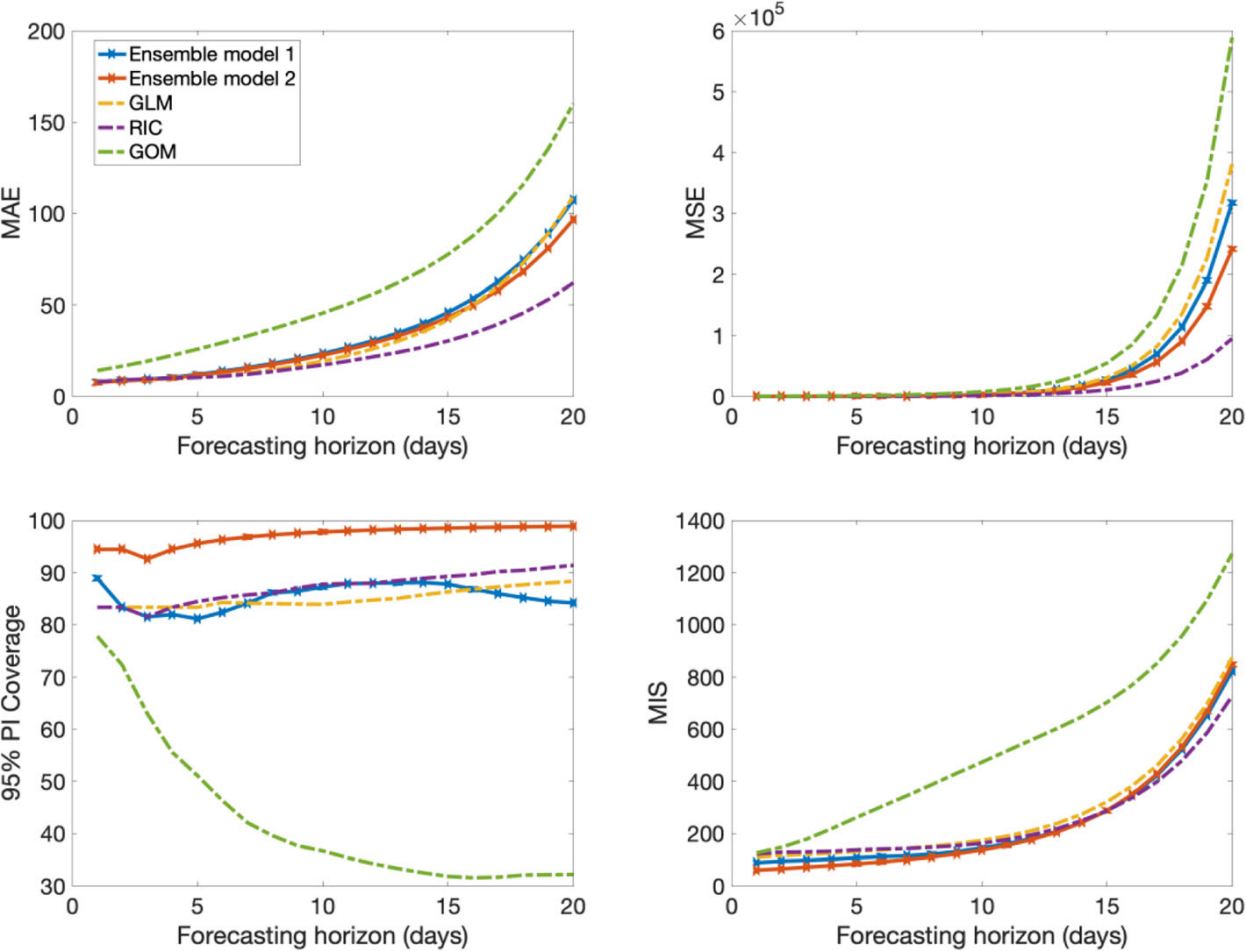
Mean performance of the individual and ensemble models in 1–20 day ahead forecasts for the COVID-19 epidemic in Guangdong. The Ensemble Method 2 outperformed all other models based on the coverage rate of the 95% PI and the MIS

**Fig. 12:**
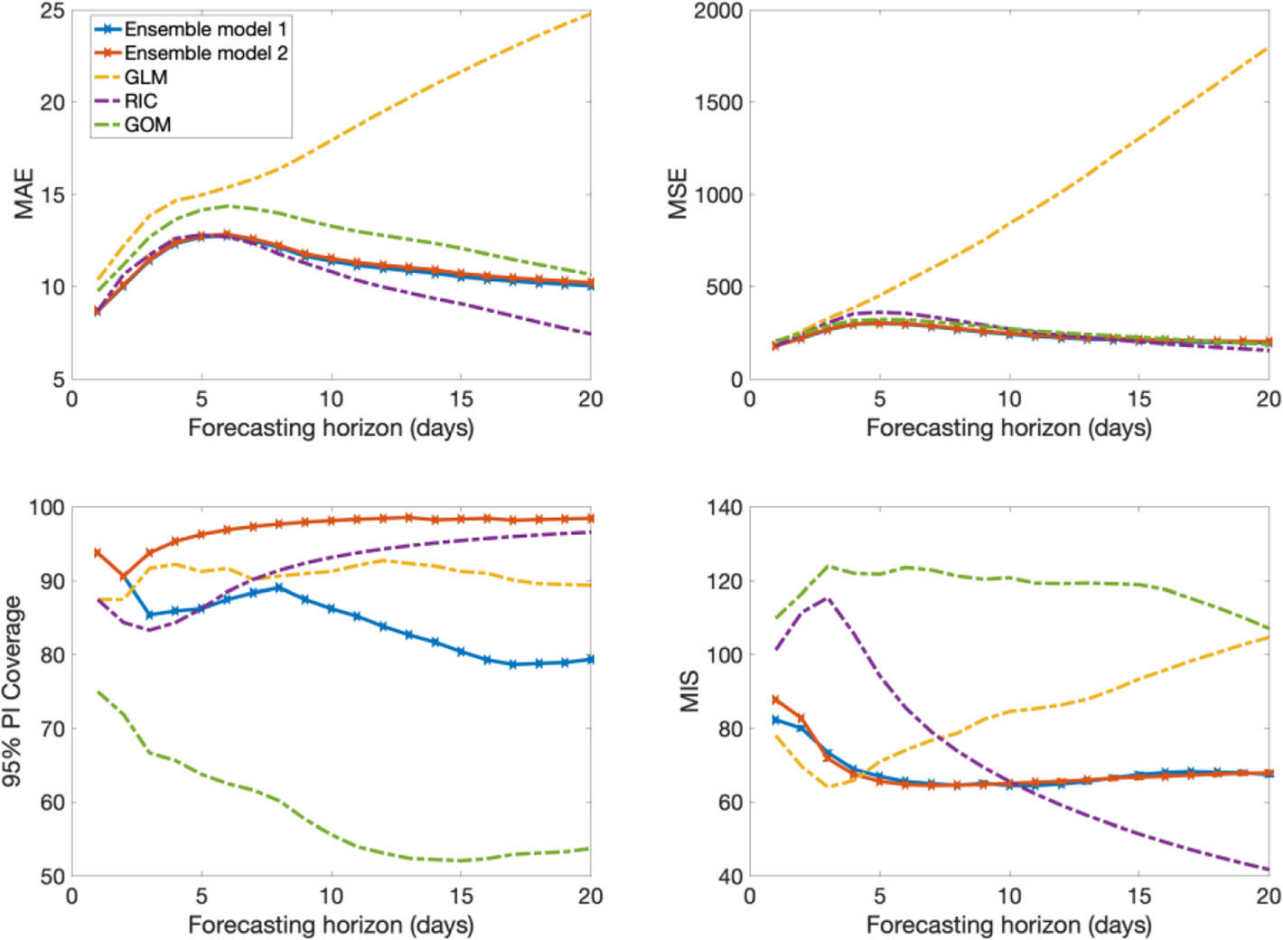
Mean performance of the individual and ensemble models in 1–20 day ahead forecasts for the COVID-19 epidemic in Henan. The Ensemble Method 2 outperformed all other models based on the coverage rate of the 95% PI and the MIS

**Fig. 13:**
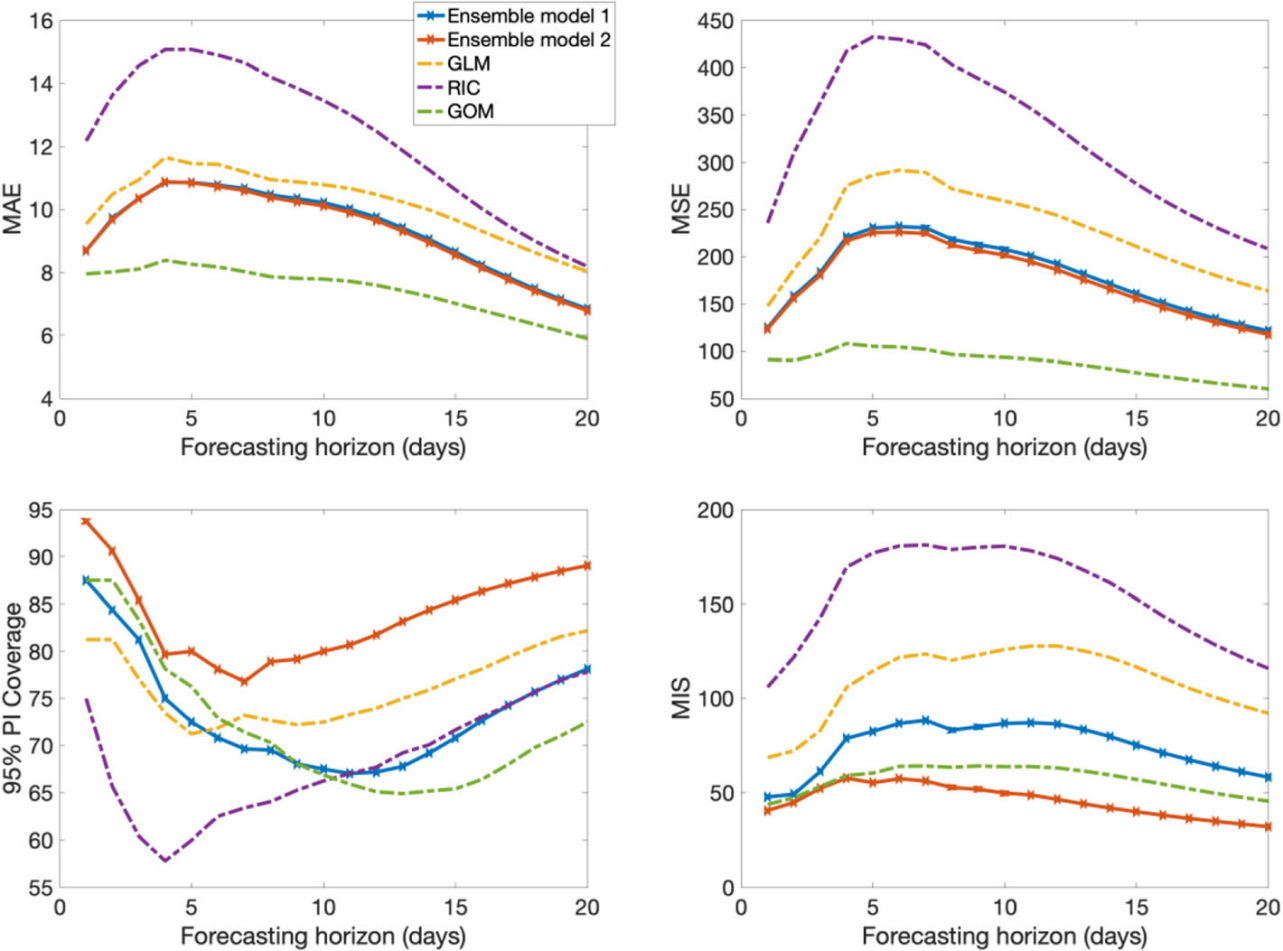
Mean performance of the individual and ensemble models in 1–20 day ahead forecasts for the COVID-19 epidemic in Hunan. The Ensemble Method 2 outperformed all other models based on the coverage rate of the 95% PI and the MIS

**Fig. 14:**
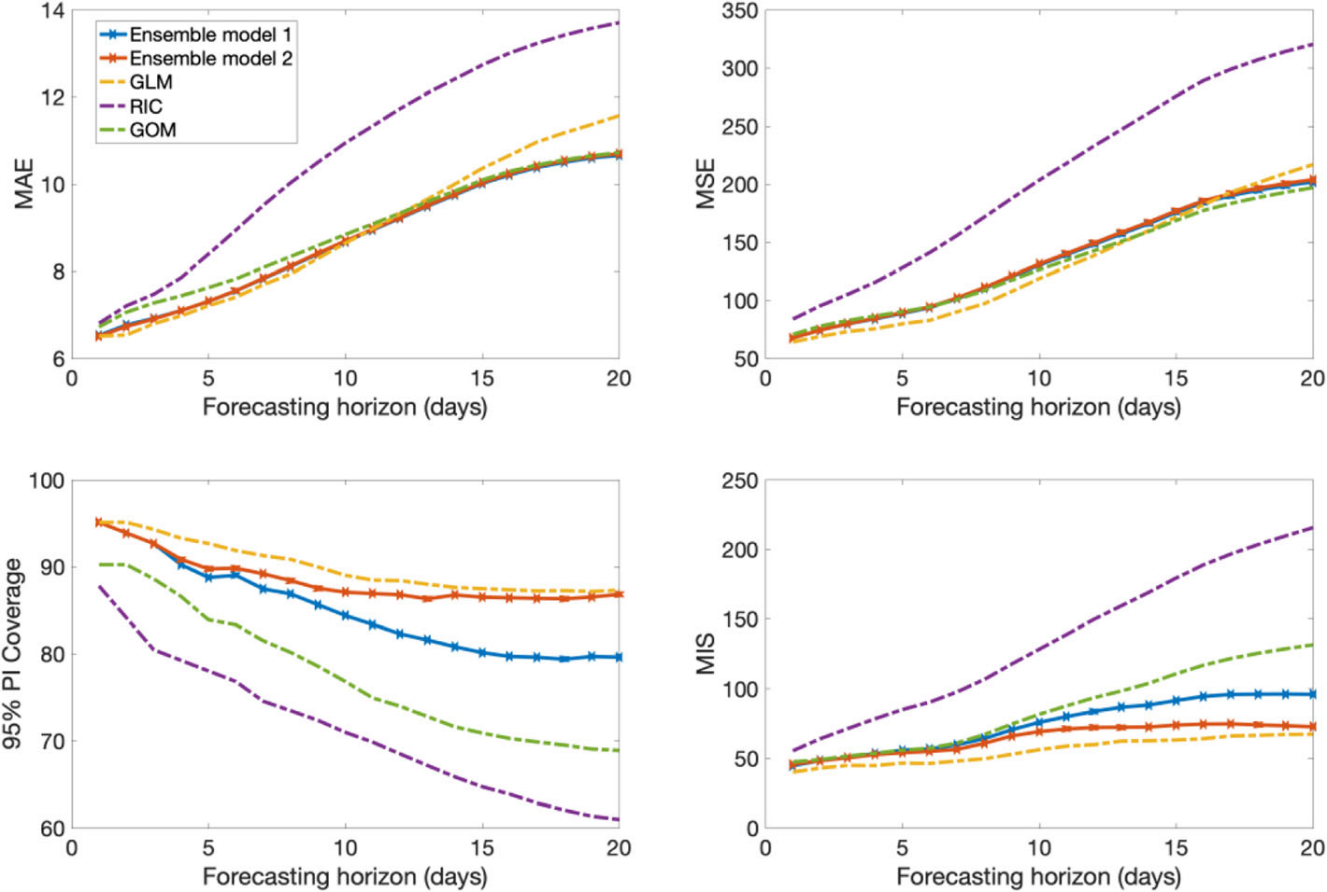
Mean performance of the individual and ensemble models in 1–20 day ahead forecasts for the 2016 Zika epidemic in Antioquia, Colombia. The GLM yields best forecasting performance in terms of the coverage rate and the MIS, but it does not achieve great advantage over the Ensemble Method 2

For real epidemic data, we found that the Ensemble Method 2 consistently yielded robust forecasting performance compared to other models according to probabilistic performance metrics (Figs. 7, 8, 9, 10, 11, 12, 13 and 14 & Figures S12, S13, S14, S15, S16, S17, S18 and S19). Specifically, for the A/H1N1 influenza epidemic in Manitoba, Canada, the plague outbreak in Madagascar, the 1918 influenza epidemic in San Francisco, the SARS outbreak in Singapore, and three COVID-19 epidemics in the Chinese provinces of Guangdong, Henan and Hunan, forecasts from the Ensemble Method 2 outperformed all other models based on the coverage rate of the 95% PI and achieved lower MIS albeit for most forecasting horizons even as individual models often attained lower MAE or MSE (i.e., which means that the predicted value is closer to the observed value). For the Zika epidemic in Antioquia, the GLM yielded best forecasting performance for all metrics, but the Ensemble Method 2 achieved similar performance (Fig. 14 and Figure S19).

## Discussion

We have introduced a simple yet-powerful methodology based on parametric bootstrapping for constructing ensemble forecasts and assessing their uncertainty from any number of individual dynamic models of variable complexity that are defined by a system of differential equations. Specifically, we introduced algorithms and assessed forecasting performance for two ensemble methods that differ in how the variance is evaluated for the generation of the prediction intervals of the forecasts. This methodology was illustrated in the context of three simple and well-known dynamical growth models with an outstanding track record in short-term epidemic forecasting [1, 4]. However, our methodology is applicable to any type of dynamic models based on differential equations ranging from phenomenological, semi-mechanistic to fully mechanistic models. We found that Ensemble Method 2 which randomly selects a model from the set of individual models for each time point of the trajectory of the epidemic frequently outcompeted the individual models as well as the alternative ensemble method based on the weighted combination of the individual models. Our results suggest that forecasting performance can be improved by combining features from multiple models across the entire trajectory of an epidemic, and the epidemic can follow or be dominated by different models at different times. In particular, Ensemble Method 2 produced broader and more realistic uncertainty bounds for the trajectory envelope and achieved not only better coverage rate of the 95% PI but also improved mean interval scores across a diversity of epidemic datasets.

Investigating different model weighting strategies to construct ensemble models is a promising direction to improve ensemble methodologies. Here we relied on the quality of the model fit to weight the individual models, but alternative strategies could be investigated. For instance, the weights could be a function of the models’ forecasting performance during previous time periods [4]. One could also consider systematic approaches to decide when to drop poor performing models from the ensemble model as the epidemic evolves. A systematic investigation to assess the effect of the weighting strategy may require a larger and more diverse set of models to identify meaningful differences in forecasting performance.

Our ensemble methodology can efficiently accommodate any combination of phenomenological, mechanistic, or quasi-mechanistic models which could describe a variety of growth processes beyond the spread of infectious disease. Further, the individual models could vary substantially in complexity in terms of the number of parameters and dynamic variables so long as the models are well calibrated to data. We have introduced ensemble algorithms that have shorter running time than other approaches that rely on knitting together the bootstrap realizations from all individual models [30]. Furthermore, it is important to note that the resulting ensembles are invariant compared to Bayesian ensemble modeling methods for which subjective choices on prior assumptions of the distributions of parameters across different models (or modeling teams) could influence posterior distributions, and in turn, the ensemble forecasts.

Probabilistic forecasts have been gaining more traction over the years. Here we rely on two performance metrics that account for the uncertainty of the predictions namely the coverage rate of the 95% PI and the mean interval score, which is a proper score that takes into account the proportion of the data that is covered by the prediction interval while penalizing for data points that fall outside the prediction interval [49]. However, these performance metrics are not exhaustive and additional performance metrics could be evaluated. We found that Ensemble Method 2 yielded the most stable performance even at longer forecasting horizons whereas the performance of the other models tended to deteriorate more rapidly over longer horizons. It is important to note that biases can arise when models are added or removed from the ensemble, which can happen in the context of forecasting competitions. Specifically, when the number of models utilized in the ensemble varies over time, the uncertainty associated with the ensemble estimates is obscured by the varying number of models considered across forecasting time points.

There is a need to establish and evaluate models and methods against a set of shared benchmarks which other models can use for comparison. New forecasting methodologies must be evaluated on well-known, diverse, and representative datasets. Here we assessed our methods in the context of a diversity of epidemic datasets including synthetic data from standard epidemic models to demonstrate method functionality as well as scenario outbreak data of the *Ebola Forecasting Challenge* [4] and real epidemic data involving a range of infectious diseases including influenza, plague, Zika, and COVID-19. Yet, there is a lack of studies that systematically assess forecasting performance using a catalogue of epidemic datasets involving multiple infectious diseases and social contexts. Therefore, we call on the research community to establish a curated data repository that includes diverse and representative epidemic datasets to systematically assess and record the performance of existing and new forecasting approaches including ensemble modeling methods.

## Supplementary Information

**Additional file 1: Figure S1.** Weekly incidence curves of the four epidemic scenarios of the *Ebola Forecasting Challenge* (blue circles). The dashed vertical lines indicate the start and end weeks of the weekly 4-week ahead forecasts. **Figure S2.** Representative sequential 20-day ahead forecasts (top to bottom panels) obtained from individual models (GLM, RIC, GOM) and two ensemble methods applied to synthetic data derived from a **stochastic SEIR model** with a population size of 100,000 and a time-dependent transmission rate (Fig. 3). Blue circles correspond to the data points. The mean fit (solid line) and 95% prediction interval (dashed lines) are also shown. The gray shaded areas help highlight differences in the 95% prediction intervals for the two ensemble methods. The vertical line separates the calibration period (left) from the forecasting period (right). **Figure S3.** Mean performance of the individual models and ensemble models in 1–20 day ahead forecasts from the synthetic data derived from **the stochastic SEIR model** with time-dependent transmission rate (Fig. 3). Our findings indicate that the Ensemble Method 2 outperformed all other models including Ensemble Method 1 based on the coverage rate of the 95% PI, which was closer to 0.95, and the MIS. Although the RIC model achieved a lower MAE and MSE at longer horizons compared to both Ensemble Methods, Ensemble Method 2 outperformed the other models including the Ensemble Method 1 based on the coverage rate and the MIS. **Figure S4.** Representative sequential 20-day ahead forecasts (top to bottom panels) obtained from individual models (GLM, RIC, GOM) and two ensemble methods applied to Scenario 1 of the *Ebola Forecasting Challenge* (Figure S1). Blue circles correspond to the data points. The mean fit (solid line) and 95% prediction interval (dashed lines) are also shown. The gray shaded areas further highlight differences in the 95% prediction intervals associated with the ensemble methods. The vertical line separates the calibration period (left) from the forecasting period (right). **Figure S5.** Mean performance of the individual and ensemble models in 1–20 day ahead forecasts from the Scenario 1 of the *Ebola Forecasting Challenge* (Figure S1). Ensemble Method 2 achieved consistently better performance across forecasting horizons compared to the Ensemble Method 1 and the individual models. **Figure S6.** Representative sequential 20-day ahead forecasts (top to bottom panels) obtained from individual models (GLM, RIC, GOM) and two ensemble methods applied to **Scenario 2** of the *Ebola Forecasting Challenge* (Figure S1). Blue circles correspond to the data points. The mean fit (solid line) and 95% prediction interval (dashed lines) are also shown. The gray shaded areas further highlight differences in the 95% prediction intervals associated with the ensemble methods. The vertical line separates the calibration period (left) from the forecasting period (right). **Figure S7.** Mean performance of the individual and ensemble models in 1–20 day ahead forecasts from the Scenario 1 of the *Ebola Forecasting Challenge* (Figure S1). Ensemble Method 2 achieved consistently better performance across forecasting horizons compared to the Ensemble Method 1 and the individual models. **Figure S8.** Representative sequential 20-day ahead forecasts (top to bottom panels) obtained from individual models (GLM, RIC, GOM) and two ensemble methods applied to **Scenario 3** of the *Ebola Forecasting Challenge* (Figure S1). Blue circles correspond to the data points. The mean fit (solid line) and 95% prediction interval (dashed lines) are also shown. The gray shaded areas further highlight differences in the 95% prediction intervals associated with the ensemble methods. The vertical line separates the calibration period (left) from the forecasting period (right). **Figure S9.** Mean performance of the individual and ensemble models in 1–20 day ahead forecasts from the Scenario 3 of the *Ebola Forecasting Challenge* (Figure S1). Ensemble Method 2 achieved consistently better performance across forecasting horizons compared to the Ensemble Method 1 and the individual models. **Figure S10.** Representative sequential 20-day ahead forecasts (top to bottom panels) obtained from individual models (GLM, RIC, GOM) and two ensemble methods applied to **Scenario 4** of the *Ebola Forecasting Challenge* (Figure S1). Blue circles correspond to the data points. The mean fit (solid red line) and 95% prediction interval (dashed lines) are also shown. The gray shaded areas further highlight differences in the 95% prediction intervals associated with the ensemble methods. The vertical line separates the calibration period (left) from the forecasting period (right). **Figure S11.** Mean performance of the individual and ensemble models in 1–20 day ahead forecasts from the **Scenario 4** of the *Ebola Forecasting Challenge* (Figure S1). Ensemble Method 2 achieved consistently better performance across forecasting horizons compared to the Ensemble Method 1 and the individual models. **Figure S12.** Representative sequential 20-day ahead forecasts (top to bottom panels) obtained from individual models (GLM, RIC, GOM) and two ensemble methods applied to **the 2009 A/H1N1 influenza pandemic in Manitoba, Canada.** Blue circles correspond to the data points. The mean fit (solid red line) and 95% prediction interval (dashed lines) are also shown. The gray shaded areas further highlight differences in the 95% prediction intervals associated with the ensemble methods. The vertical line separates the calibration period (left) from the forecasting period (right). **Figure S13.** Representative sequential 20-day ahead forecasts (top to bottom panels) obtained from individual models (GLM, RIC, GOM) and two ensemble methods applied to **1918 influenza pandemic in San Francisco.** Blue circles correspond to the data points. The mean fit (solid line) and 95% prediction interval (dashed lines) are also shown. The gray shaded areas further highlight differences in the 95% prediction intervals associated with the ensemble methods. The vertical line separates the calibration period (left) from the forecasting period (right). **Figure S14.** Representative sequential 20-day ahead forecasts (top to bottom panels) obtained from individual models (GLM, RIC, GOM) and two ensemble methods applied to **plague epidemic in Madagascar.** Blue circles correspond to the data points. The mean fit (solid line) and 95% prediction interval (dashed lines) are also shown. The gray shaded areas further highlight differences in the 95% prediction intervals associated with the ensemble methods. The vertical line separates the calibration period (left) from the forecasting period (right). **Figure S15.** Representative sequential 20-day ahead forecasts (top to bottom panels) obtained from individual models (GLM, RIC, GOM) and two ensemble methods applied to **2003 SARS outbreak in Singapore.** Blue circles correspond to the data points. The mean fit (solid line) and 95% prediction interval (dashed lines) are also shown. The gray shaded areas further highlight differences in the 95% prediction intervals associated with the ensemble methods. The vertical line separates the calibration period (left) from the forecasting period (right). **Figure S16.** Representative sequential 20-day ahead forecasts (top to bottom panels) obtained from individual models (GLM, RIC, GOM) and two ensemble methods applied to **the COVID-19 epidemic in Guangdong.** Blue circles correspond to the data points. The mean fit (solid line) and 95% prediction interval (dashed lines) are also shown. The gray shaded areas further highlight differences in the 95% prediction intervals associated with the ensemble methods. The vertical line separates the calibration period (left) from the forecasting period (right). **Figure S17.** Representative sequential 20-day ahead forecasts (top to bottom panels) obtained from individual models (GLM, RIC, GOM) and two ensemble methods applied to **the COVID-19 epidemic in Henan.** Blue circles correspond to the data points. The mean fit (solid line) and 95% prediction interval (dashed lines) are also shown. The gray shaded areas further highlight differences in the 95% prediction intervals associated with the ensemble methods. The vertical line separates the calibration period (left) from the forecasting period (right). **Figure S18.** Representative sequential 20-day ahead forecasts (top to bottom to panels) obtained from individual models (GLM, RIC, GOM) and two ensemble methods applied to **the COVID-19 epidemic in Hunan.** Blue circles correspond to the data points. The mean fit (solid line) and 95% prediction interval (dashed lines) are also shown. The gray shaded areas further highlight differences in the 95% prediction intervals associated with the ensemble methods. The vertical line separates the calibration period (left) from the forecasting period (right). **Figure S19.** Representative sequential 20-day ahead forecasts (top to bottom panels) obtained from individual models (GLM, RIC, GOM) and two ensemble methods applied to the Zika epidemic in Antioquia, Colombia. Blue circles correspond to the data points. The mean fit (solid line) and 95% prediction interval (dashed lines) are also shown. The gray shaded areas further highlight differences in the 95% prediction intervals associated with the ensemble methods. The vertical line separates the calibration period (left) from the forecasting period (right).

## Data Availability

All of the data are publicly available in ref. [55].
