# Supplementary material for: Ensemble bootstrap methodology for forecasting dynamic growth processes using differential equations: application to epidemic outbreaks
Source: BMC Med Res Methodol. 2021 Feb 14;21:34. doi: 10.1186/s12874-021-01226-9 (PMC7882252; doi:10.1186/s12874-021-01226-9)

**Supplement**

**Ensemble bootstrap methodology for forecasting dynamic growth processes using differential equations: Application to epidemic outbreaks**

Gerardo Chowell^1,2,*^ & Ruiyan Luo^1^

^1^ Department of Population Heath Sciences, School of Public Health, Georgia State University, Atlanta, GA, USA

^2^ Division of International Epidemiology and Population Studies, Fogarty International Center, National Institutes of Health, Bethesda, MD, USA

^*^ Corresponding author ([gchowell@gsu.edu](mailto:gchowell@gsu.edu))

**Figure S1.** Weekly incidence curves of the four epidemic scenarios of the *Ebola Forecasting Challenge* (blue circles). The dashed vertical lines indicate the start and end weeks of the weekly 4-week ahead forecasts.


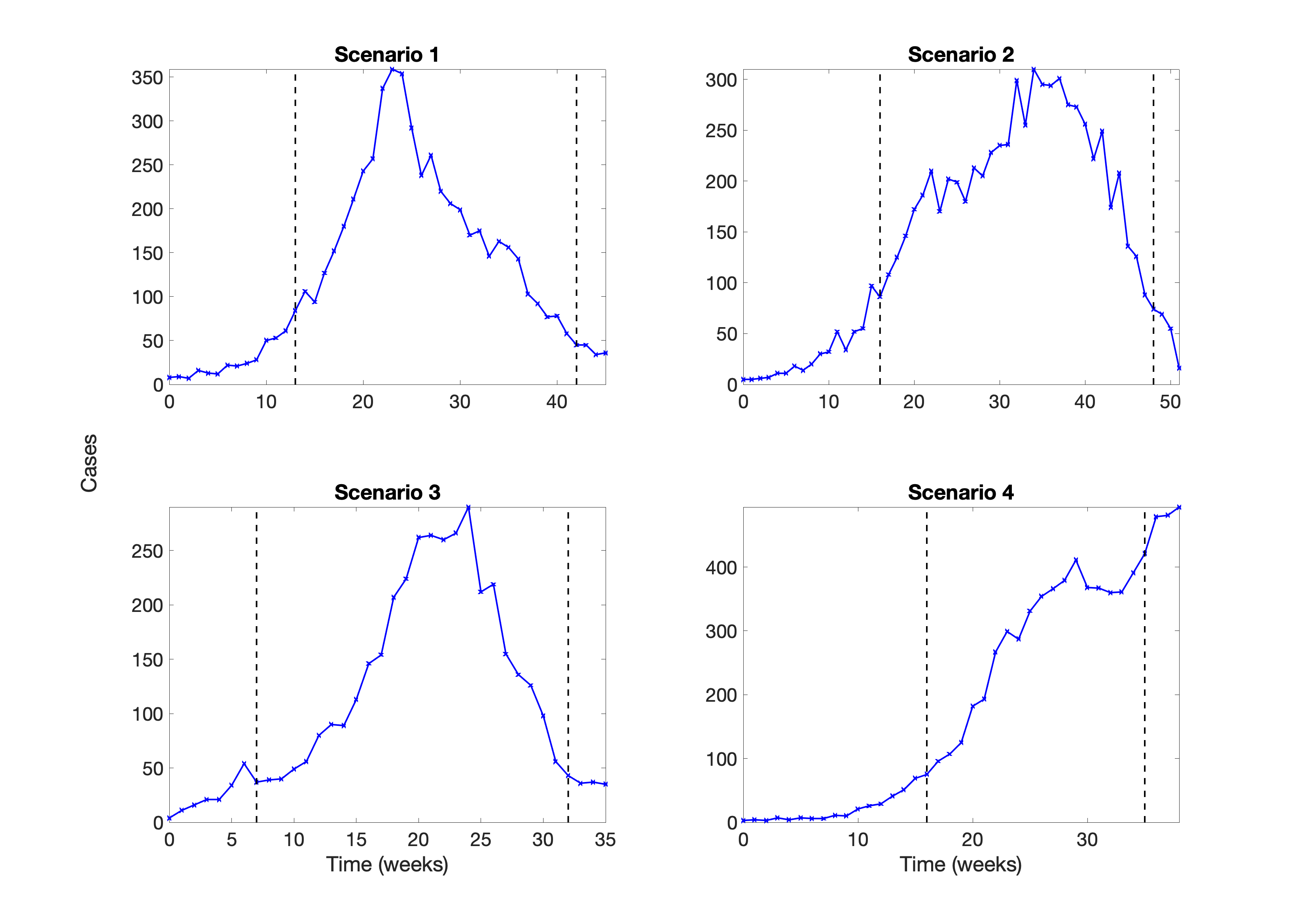


**Figure S2**. Representative sequential 20-day ahead forecasts (top to bottom panels) obtained from individual models (GLM, RIC, GOM) and two ensemble methods applied to synthetic data derived from a **stochastic SEIR model** with a population size of 100,000 and a time-dependent transmission rate (Figure 3). Blue circles correspond to the data points. The mean fit (solid line) and 95% prediction interval (dashed lines) are also shown. The gray shaded areas help highlight differences in the 95% prediction intervals for the two ensemble methods. The vertical line separates the calibration period (left) from the forecasting period (right).


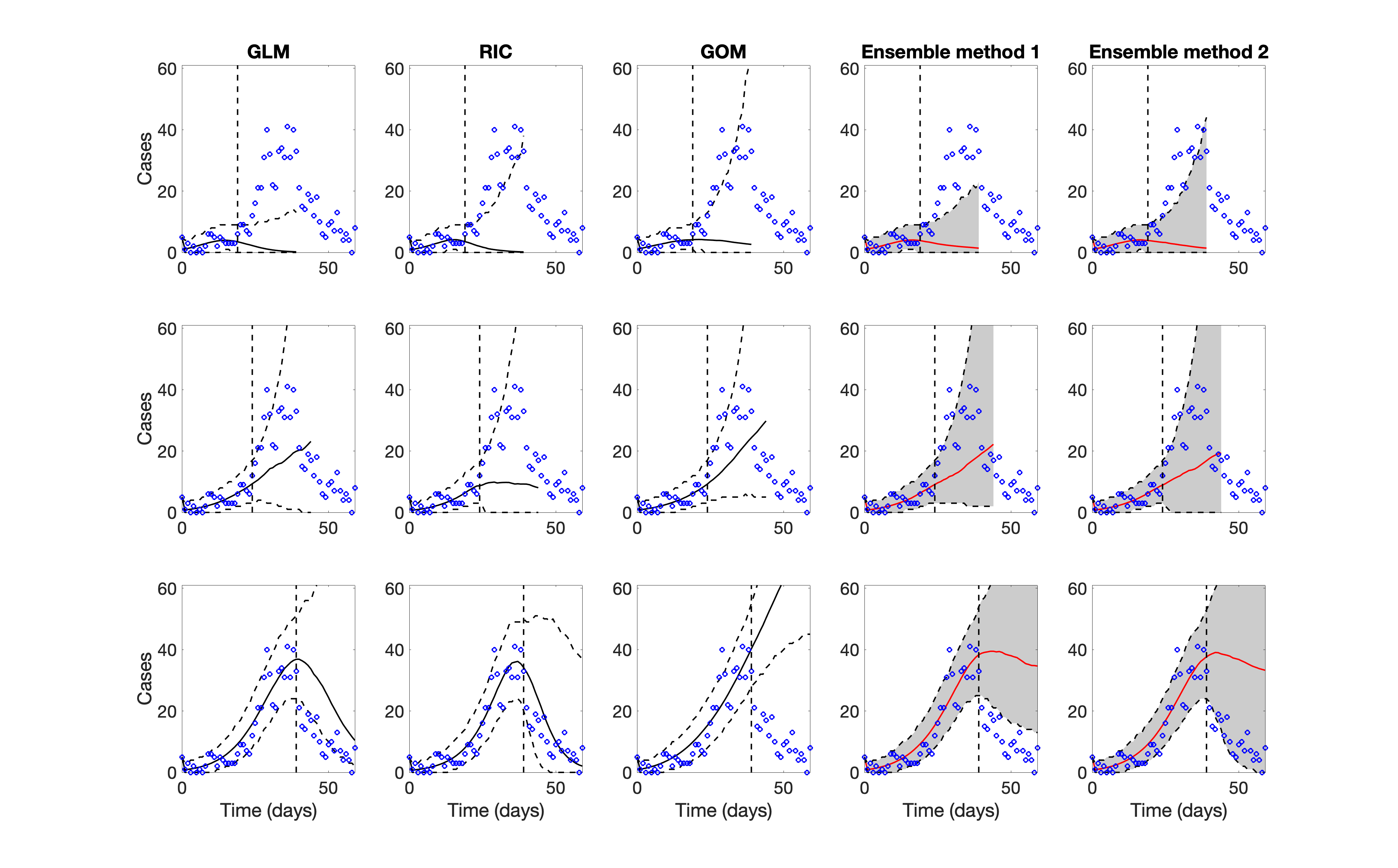


**Figure S3.** Mean performance of the individual models and ensemble models in 1-20 day ahead forecasts from the synthetic data derived from **the stochastic SEIR model** with time-dependent transmission rate (Figure 3). Our findings indicate that the Ensemble Method 2 outperformed all other models including Ensemble Method 1 based on the coverage rate of the 95% PI, which was closer to 0.95, and the MIS. Although the RIC model achieved a lower MAE and MSE at longer horizons compared to both Ensemble Methods, Ensemble Method 2 outperformed the other models including the Ensemble Method 1 based on the coverage rate and the MIS.


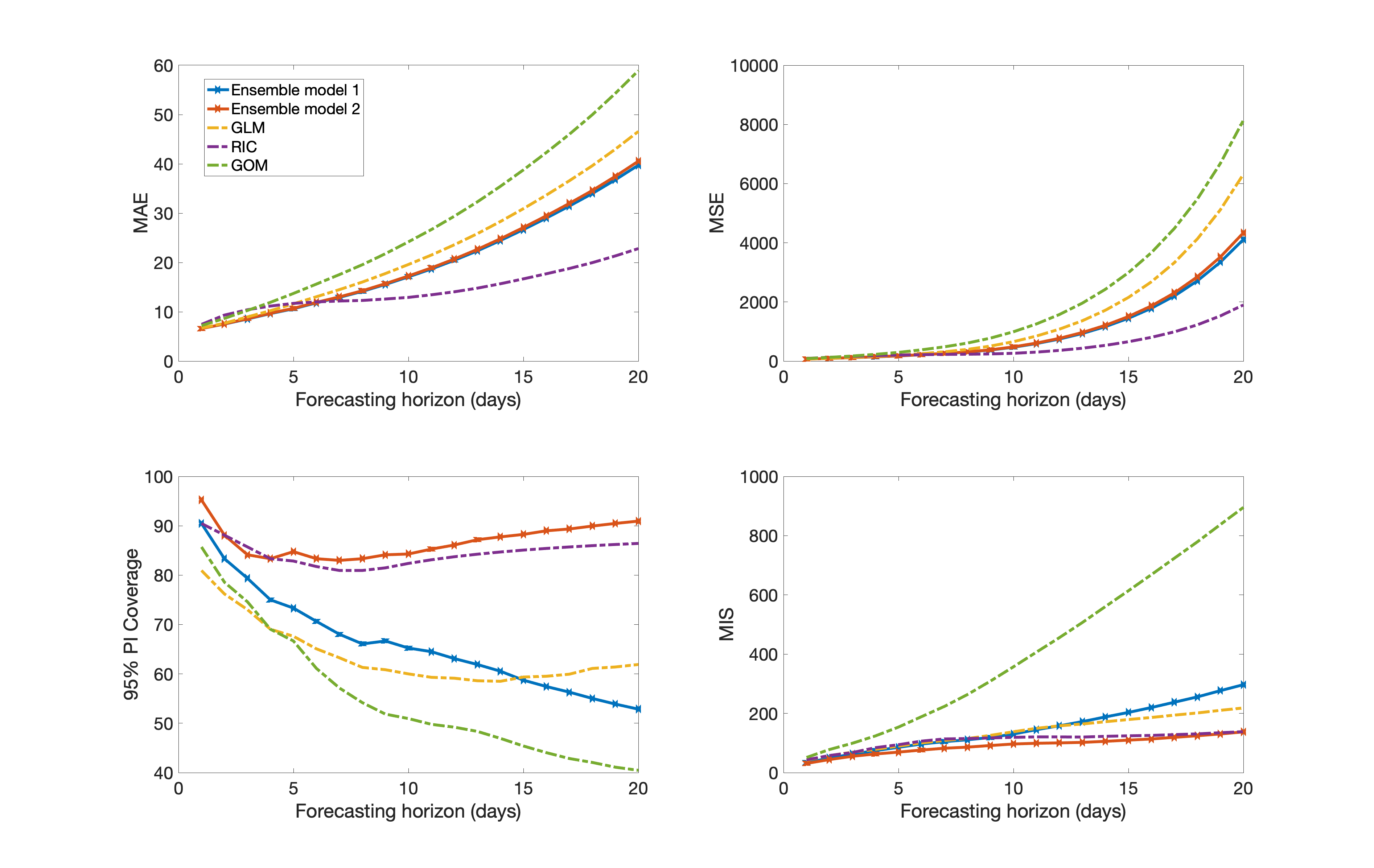


**Figure S4**. Representative sequential 20-day ahead forecasts (top to bottom panels) obtained from individual models (GLM, RIC, GOM) and two ensemble methods applied to Scenario 1 of the *Ebola Forecasting Challenge* (Figure S1). Blue circles correspond to the data points. The mean fit (solid line) and 95% prediction interval (dashed lines) are also shown. The gray shaded areas further highlight differences in the 95% prediction intervals associated with the ensemble methods. The vertical line separates the calibration period (left) from the forecasting period (right).


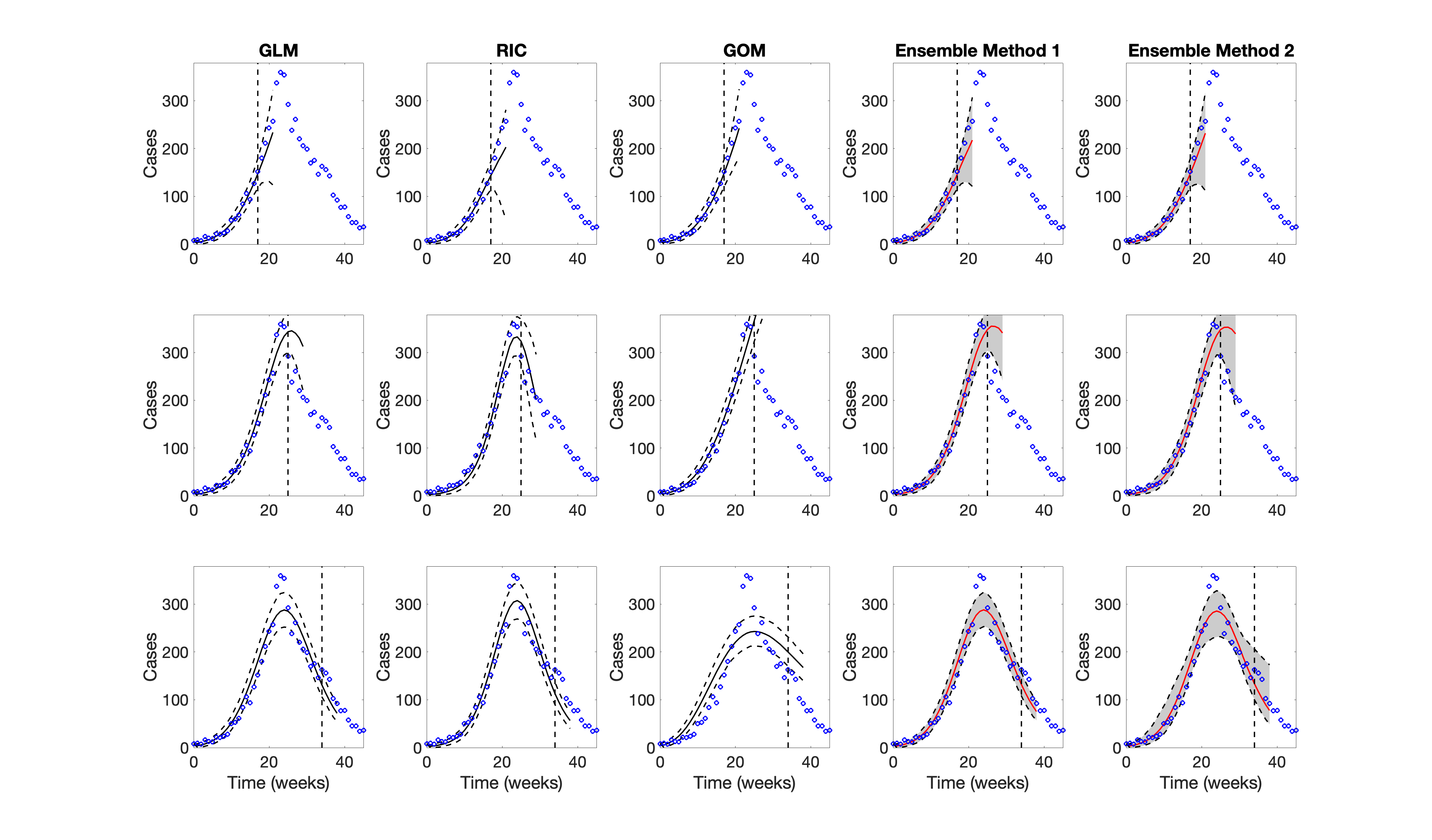


**Figure S5**. Mean performance of the individual and ensemble models in 1-20 day ahead forecasts from the Scenario 1 of the *Ebola Forecasting Challenge* (Figure S1). Ensemble Method 2 achieved consistently better performance across forecasting horizons compared to the Ensemble Method 1 and the individual models.


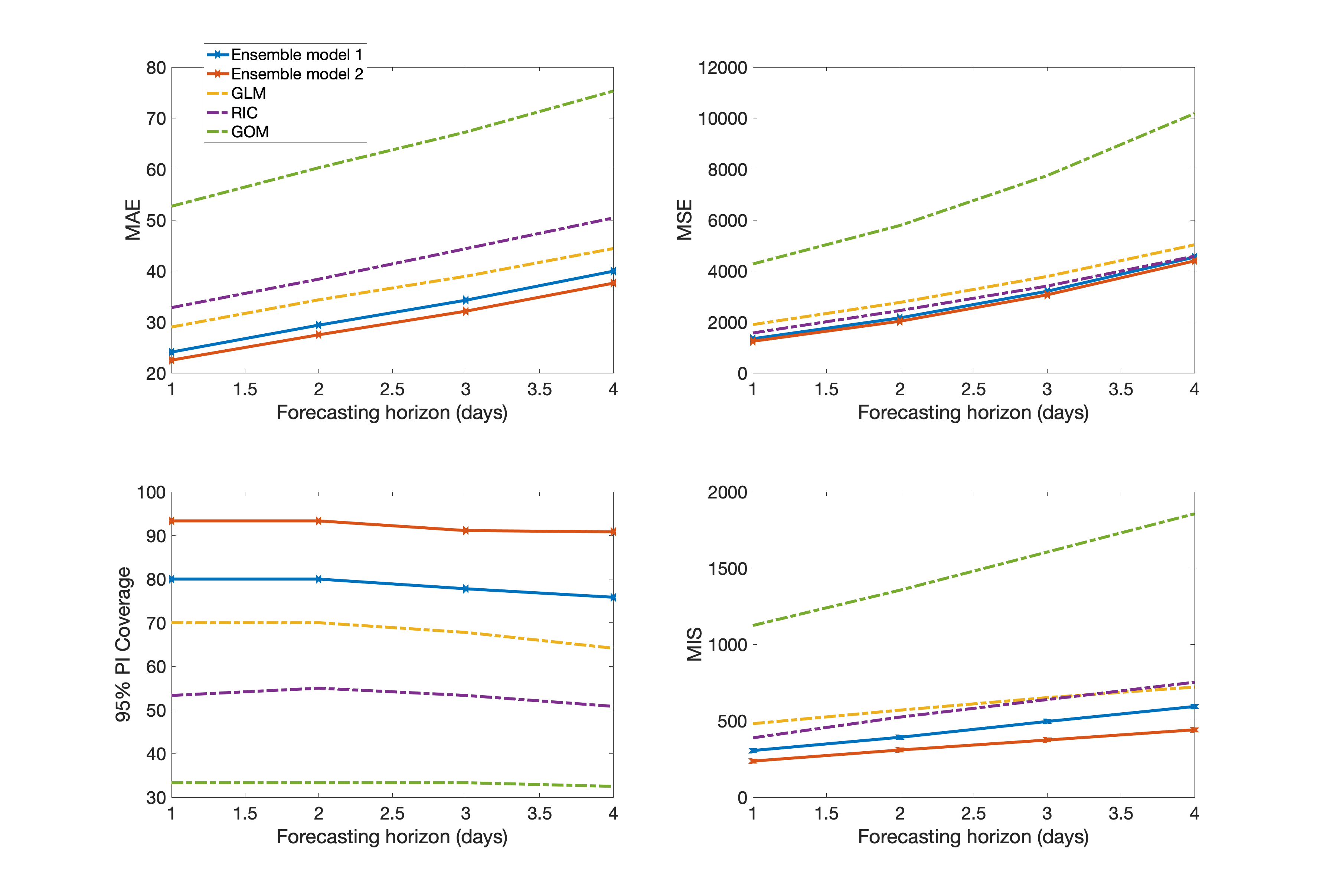


**Figure S6**. Representative sequential 20-day ahead forecasts (top to bottom panels) obtained from individual models (GLM, RIC, GOM) and two ensemble methods applied to **Scenario 2** of the *Ebola Forecasting Challenge* (Figure S1). Blue circles correspond to the data points. The mean fit (solid line) and 95% prediction interval (dashed lines) are also shown. The gray shaded areas further highlight differences in the 95% prediction intervals associated with the ensemble methods. The vertical line separates the calibration period (left) from the forecasting period (right).


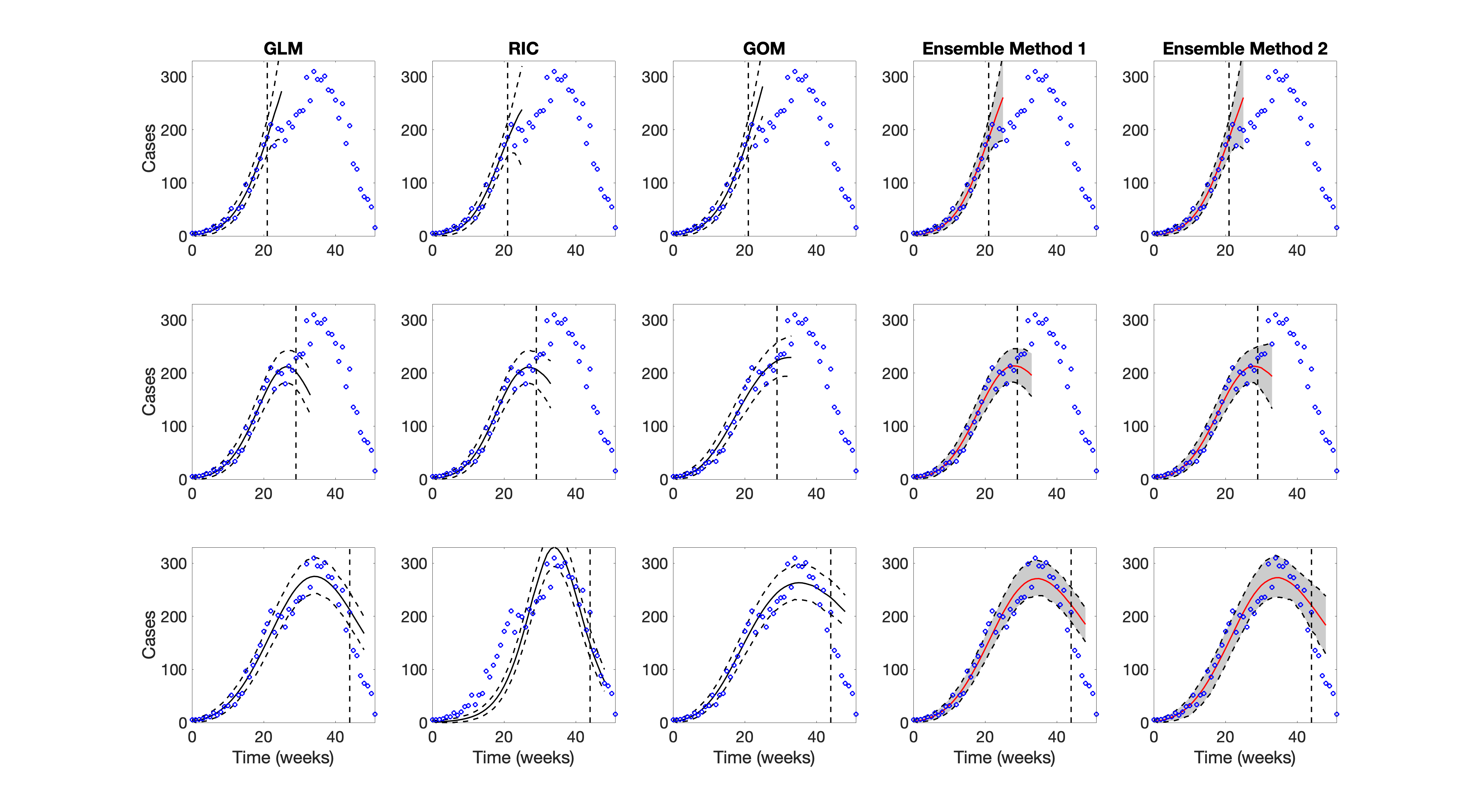


**Figure S7**. Mean performance of the individual and ensemble models in 1-20 day ahead forecasts from the Scenario 1 of the *Ebola Forecasting Challenge* (Figure S1). Ensemble Method 2 achieved consistently better performance across forecasting horizons compared to the Ensemble Method 1 and the individual models.


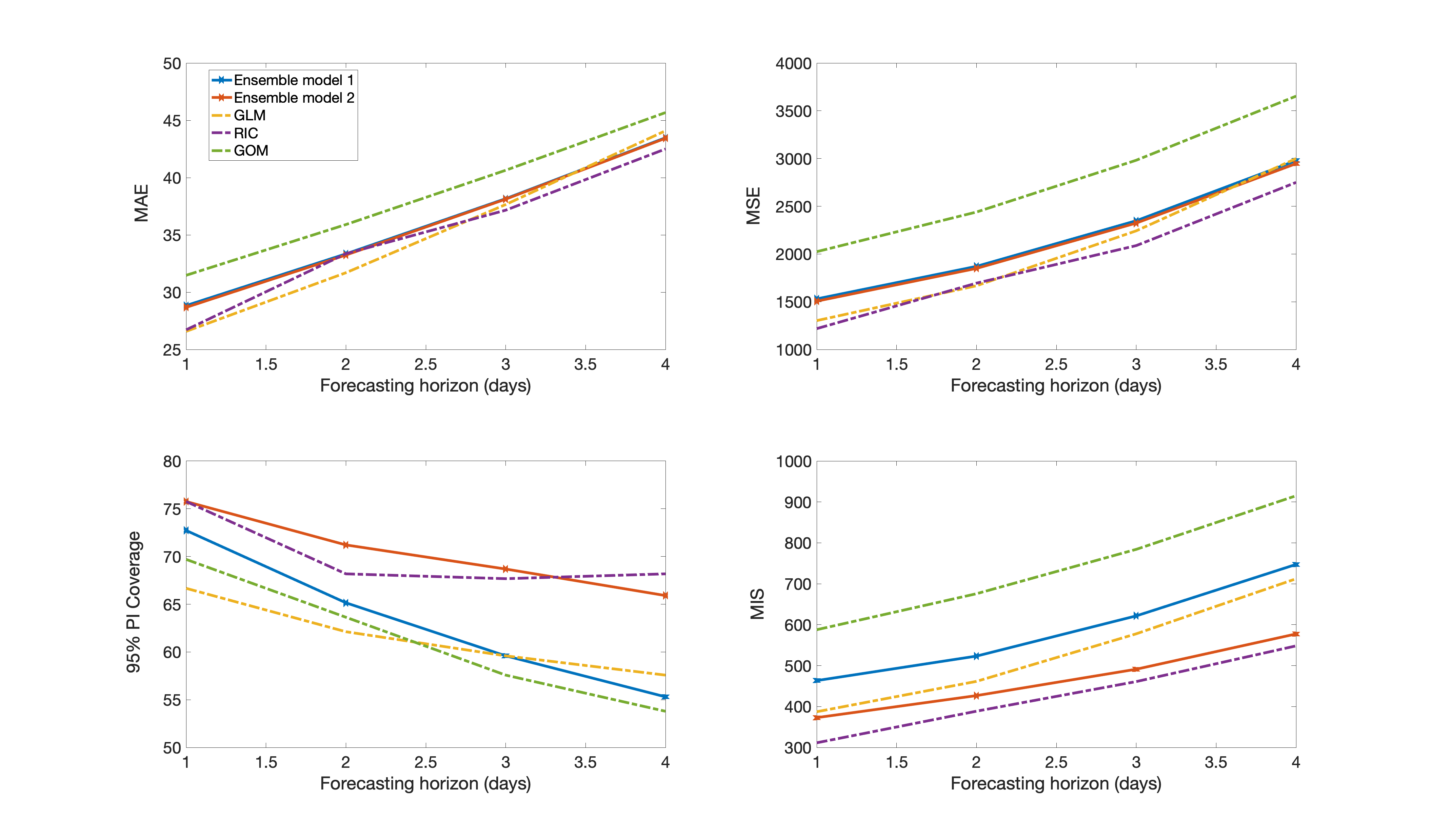


**Figure S8**. Representative sequential 20-day ahead forecasts (top to bottom panels) obtained from individual models (GLM, RIC, GOM) and two ensemble methods applied to **Scenario 3** of the *Ebola Forecasting Challenge* (Figure S1). Blue circles correspond to the data points. The mean fit (solid line) and 95% prediction interval (dashed lines) are also shown. The gray shaded areas further highlight differences in the 95% prediction intervals associated with the ensemble methods. The vertical line separates the calibration period (left) from the forecasting period (right).


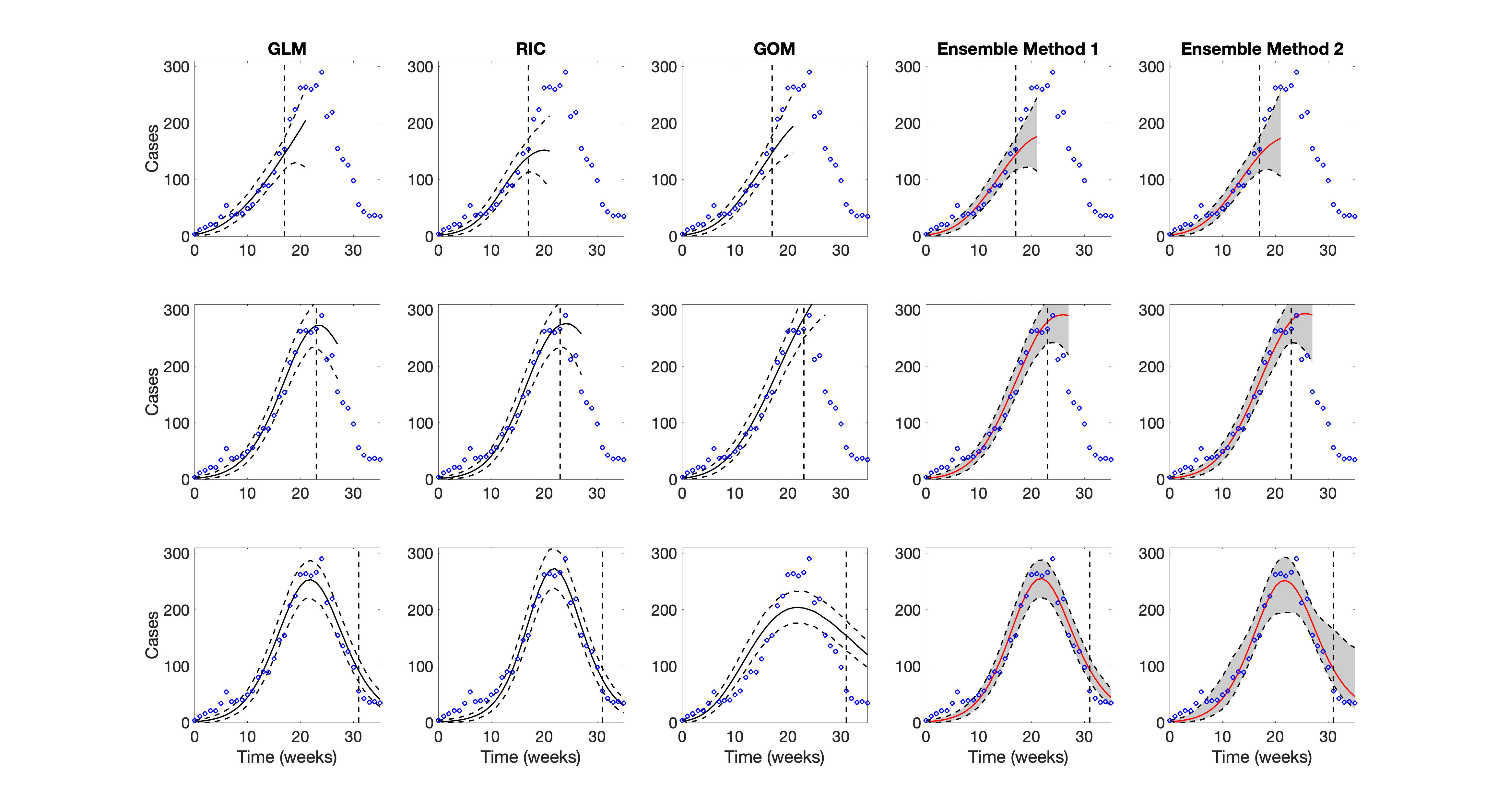


**Figure S9**. Mean performance of the individual and ensemble models in 1-20 day ahead forecasts from the Scenario 3 of the *Ebola Forecasting Challenge* (Figure S1). Ensemble Method 2 achieved consistently better performance across forecasting horizons compared to the Ensemble Method 1 and the individual models.


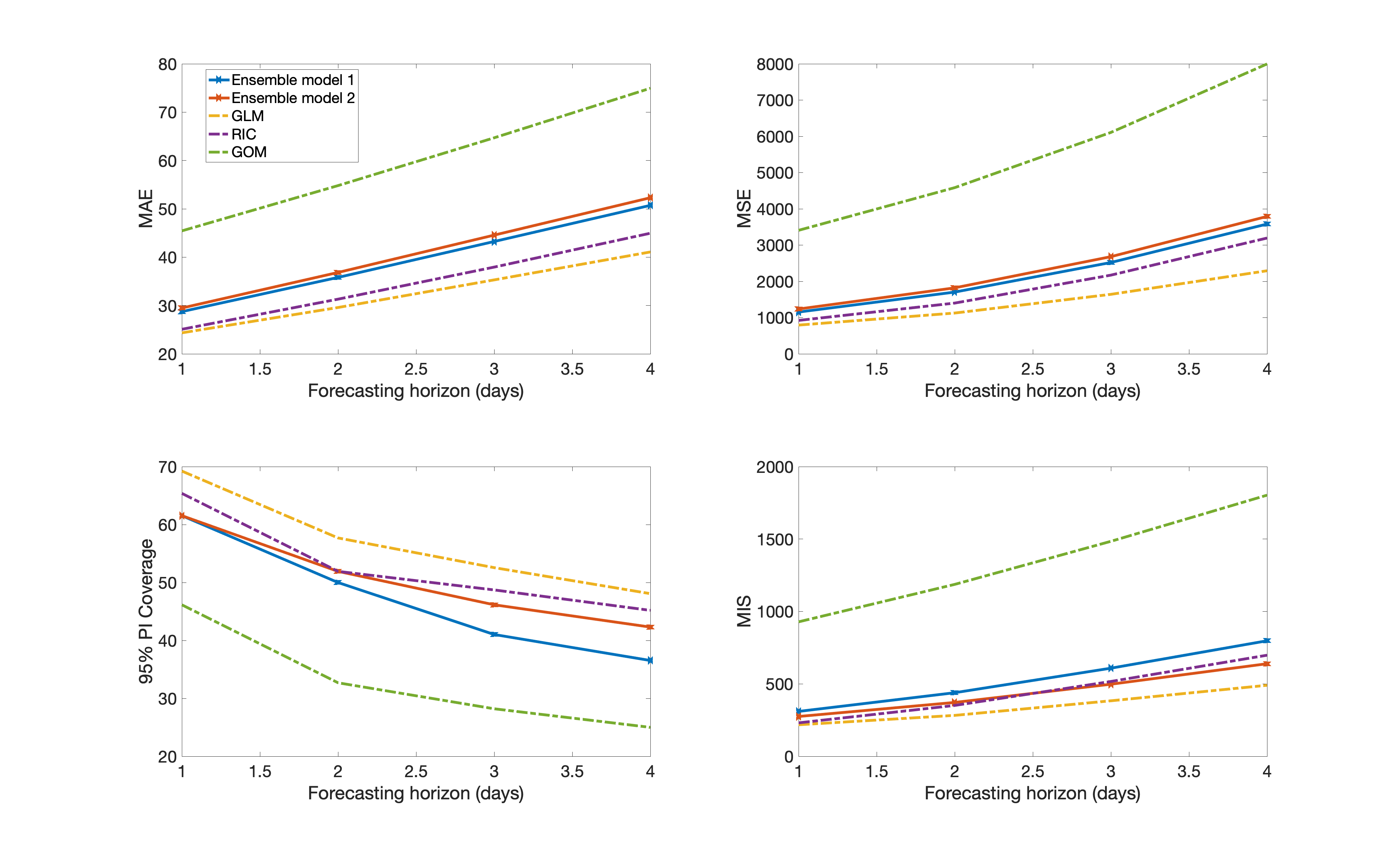


**Figure S10**. Representative sequential 20-day ahead forecasts (top to bottom panels) obtained from individual models (GLM, RIC, GOM) and two ensemble methods applied to **Scenario 4** of the *Ebola Forecasting Challenge* (Figure S1). Blue circles correspond to the data points. The mean fit (solid red line) and 95% prediction interval (dashed lines) are also shown. The gray shaded areas further highlight differences in the 95% prediction intervals associated with the ensemble methods. The vertical line separates the calibration period (left) from the forecasting period (right).


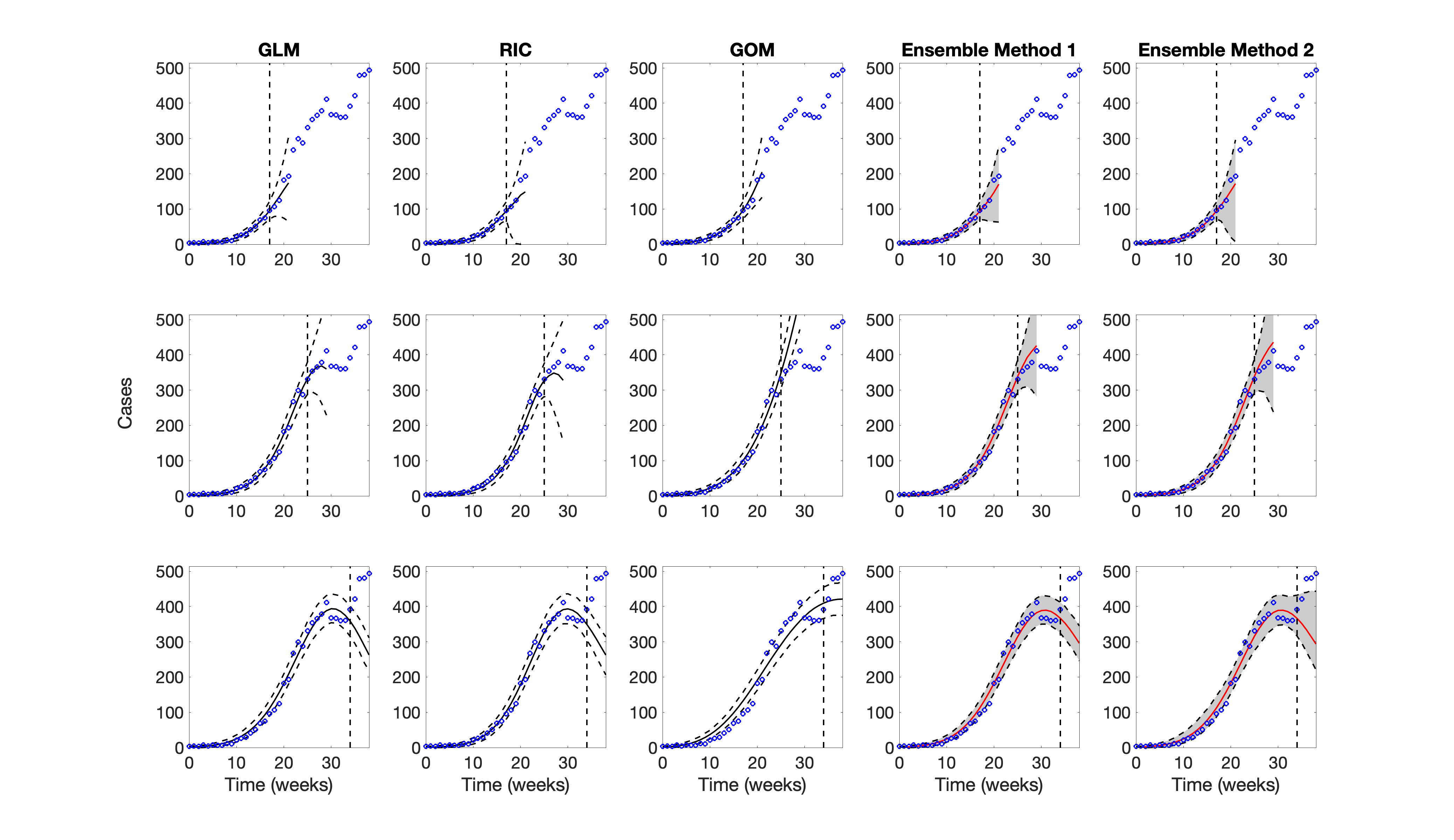


**Figure S11**. Mean performance of the individual and ensemble models in 1-20 day ahead forecasts from the **Scenario 4** of the *Ebola Forecasting Challenge* (Figure S1). Ensemble Method 2 achieved consistently better performance across forecasting horizons compared to the Ensemble Method 1 and the individual models.


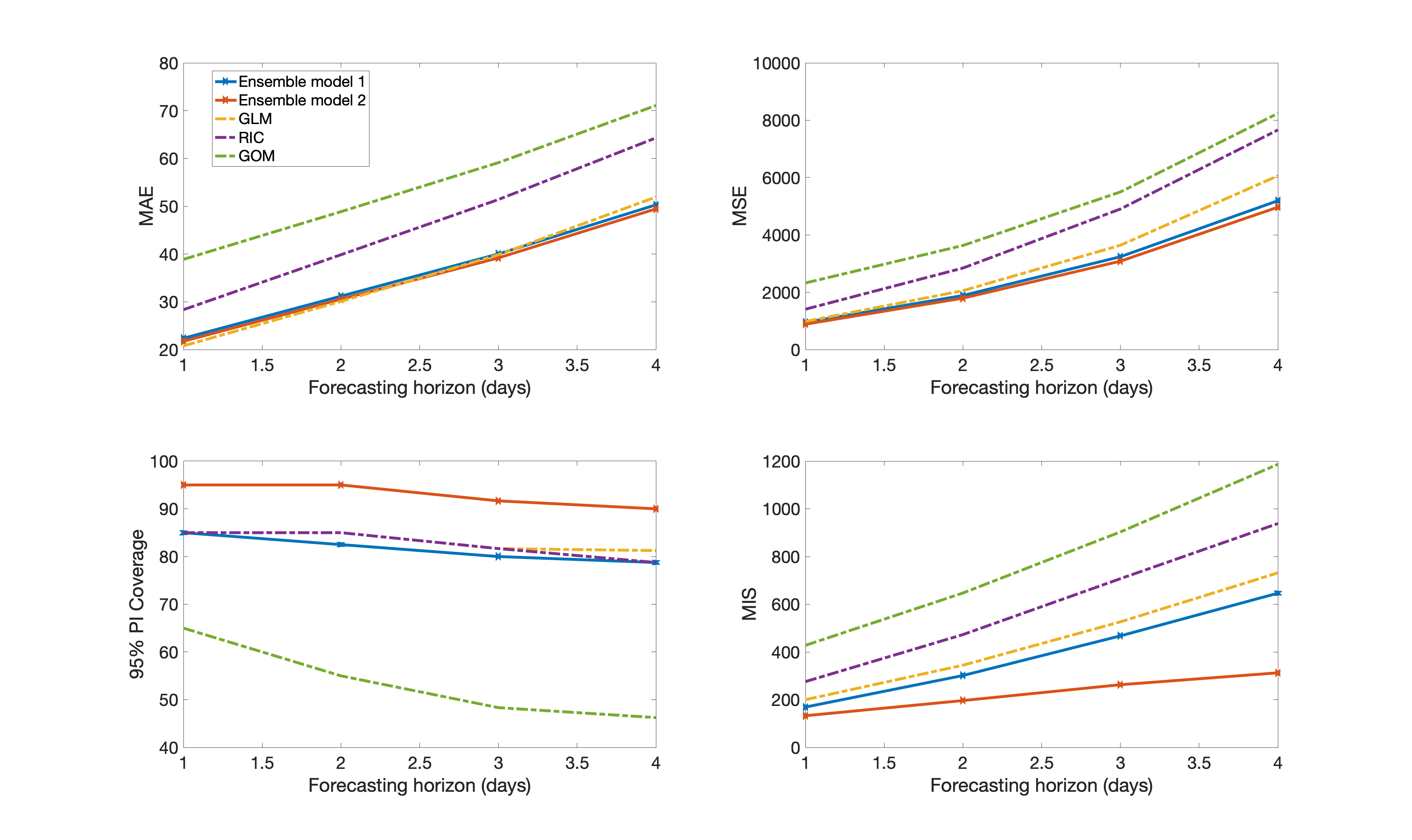


**Figure S12**. Representative sequential 20-day ahead forecasts (top to bottom panels) obtained from individual models (GLM, RIC, GOM) and two ensemble methods applied to **the 2009 A/H1N1 influenza pandemic in Manitoba, Canada.** Blue circles correspond to the data points. The mean fit (solid red line) and 95% prediction interval (dashed lines) are also shown. The gray shaded areas further highlight differences in the 95% prediction intervals associated with the ensemble methods. The vertical line separates the calibration period (left) from the forecasting period (right).


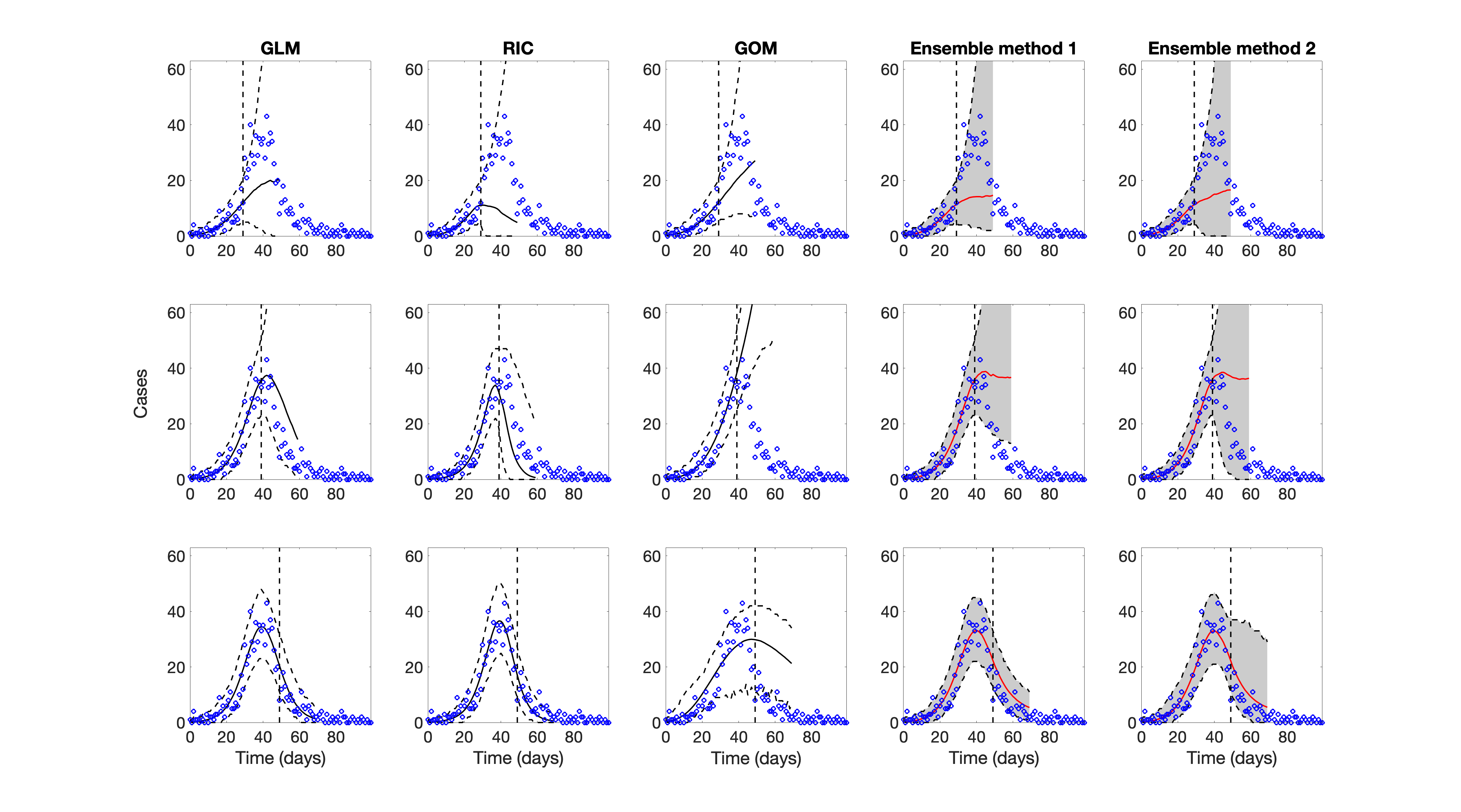


**Figure S13**. Representative sequential 20-day ahead forecasts (top to bottom panels) obtained from individual models (GLM, RIC, GOM) and two ensemble methods applied to **1918 influenza pandemic in San Francisco.** Blue circles correspond to the data points. The mean fit (solid line) and 95% prediction interval (dashed lines) are also shown. The gray shaded areas further highlight differences in the 95% prediction intervals associated with the ensemble methods. The vertical line separates the calibration period (left) from the forecasting period (right).


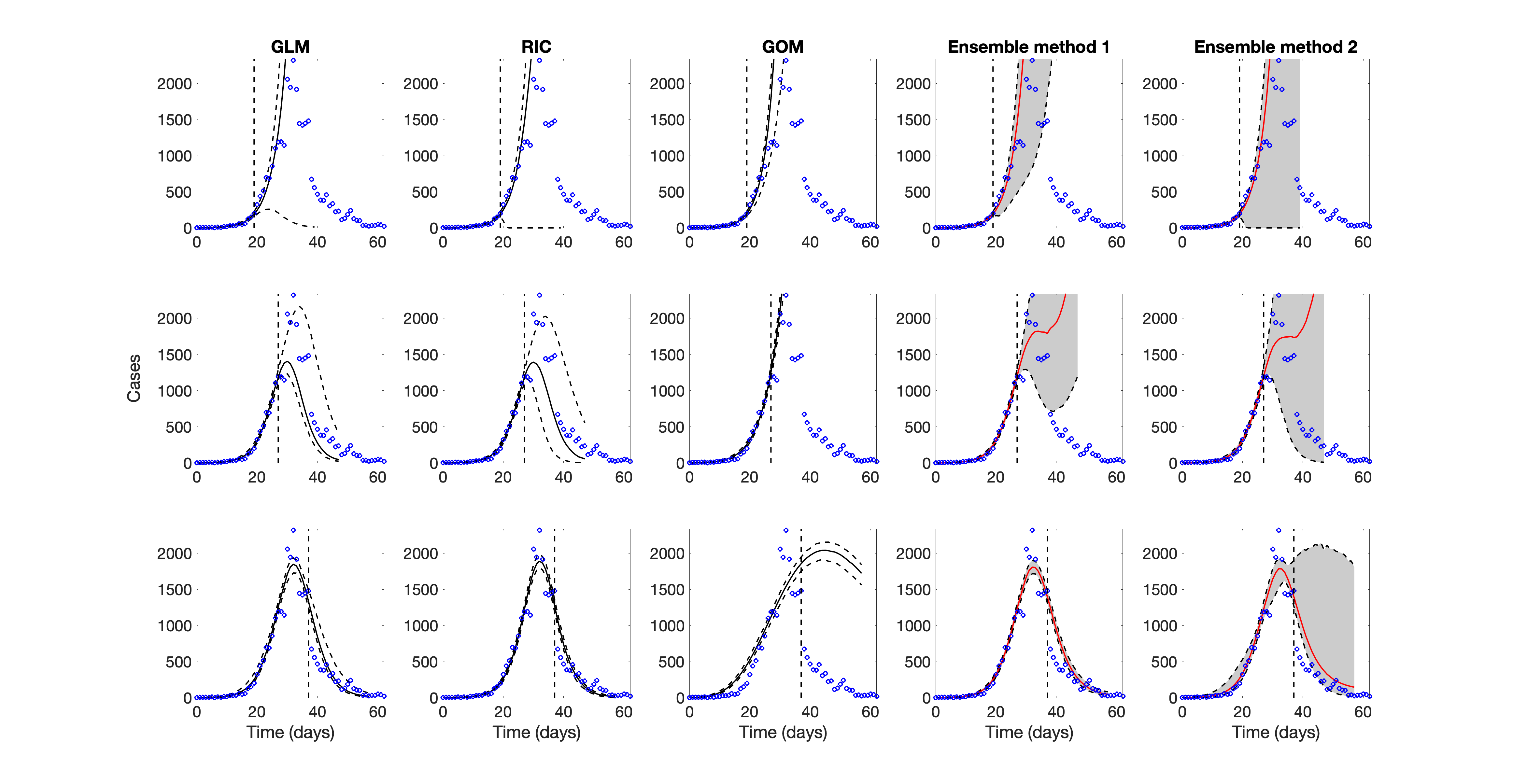


**Figure S14**. Representative sequential 20-day ahead forecasts (top to bottom panels) obtained from individual models (GLM, RIC, GOM) and two ensemble methods applied to **plague epidemic in Madagascar.** Blue circles correspond to the data points. The mean fit (solid line) and 95% prediction interval (dashed lines) are also shown. The gray shaded areas further highlight differences in the 95% prediction intervals associated with the ensemble methods. The vertical line separates the calibration period (left) from the forecasting period (right).


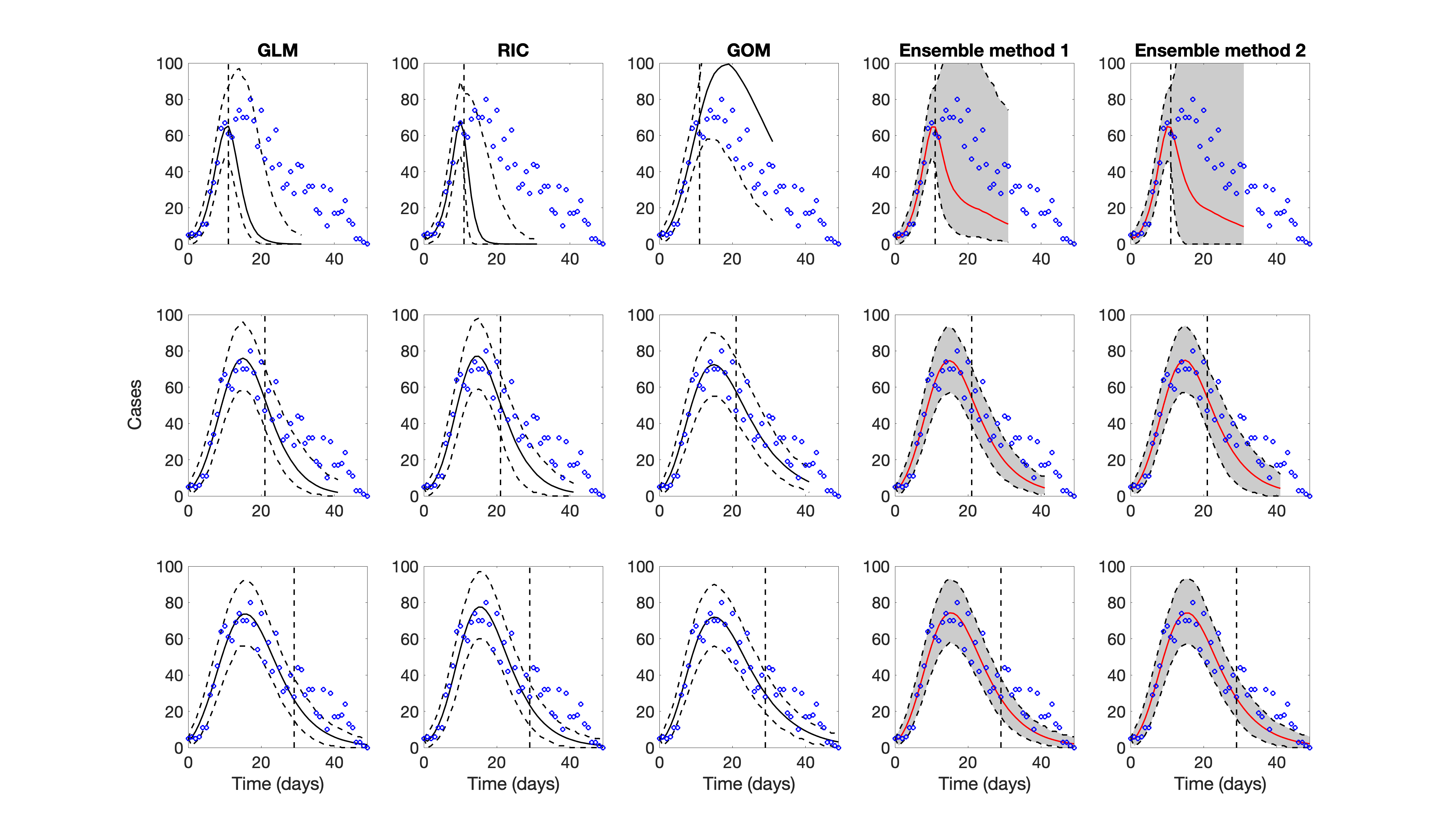


**Figure S15**. Representative sequential 20-day ahead forecasts (top to bottom panels) obtained from individual models (GLM, RIC, GOM) and two ensemble methods applied to **2003 SARS outbreak in Singapore.** Blue circles correspond to the data points. The mean fit (solid line) and 95% prediction interval (dashed lines) are also shown. The gray shaded areas further highlight differences in the 95% prediction intervals associated with the ensemble methods. The vertical line separates the calibration period (left) from the forecasting period (right).


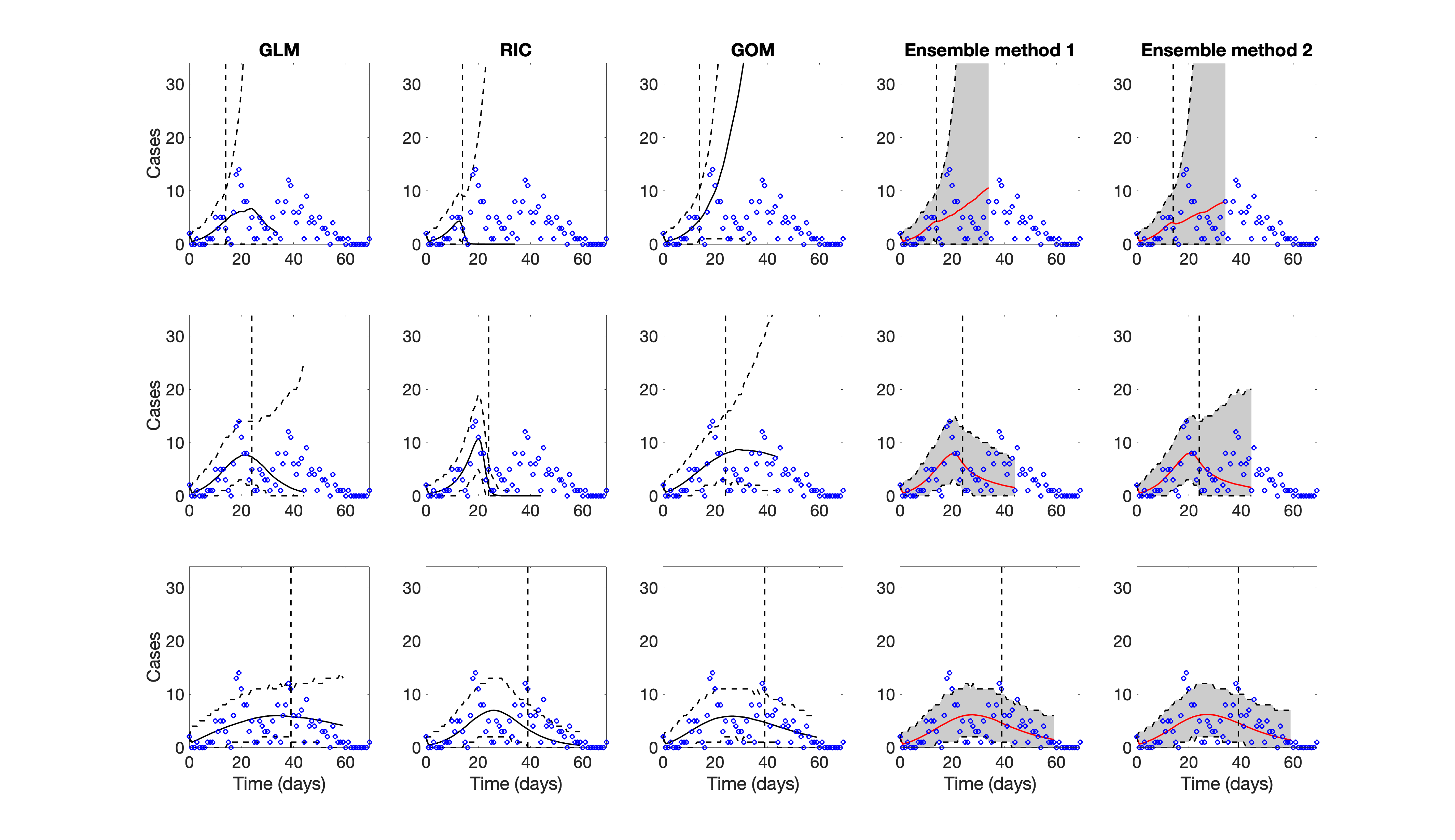


**Figure S16**. Representative sequential 20-day ahead forecasts (top to bottom panels) obtained from individual models (GLM, RIC, GOM) and two ensemble methods applied to **the COVID-19 epidemic in Guangdong.** Blue circles correspond to the data points. The mean fit (solid line) and 95% prediction interval (dashed lines) are also shown. The gray shaded areas further highlight differences in the 95% prediction intervals associated with the ensemble methods. The vertical line separates the calibration period (left) from the forecasting period (right).


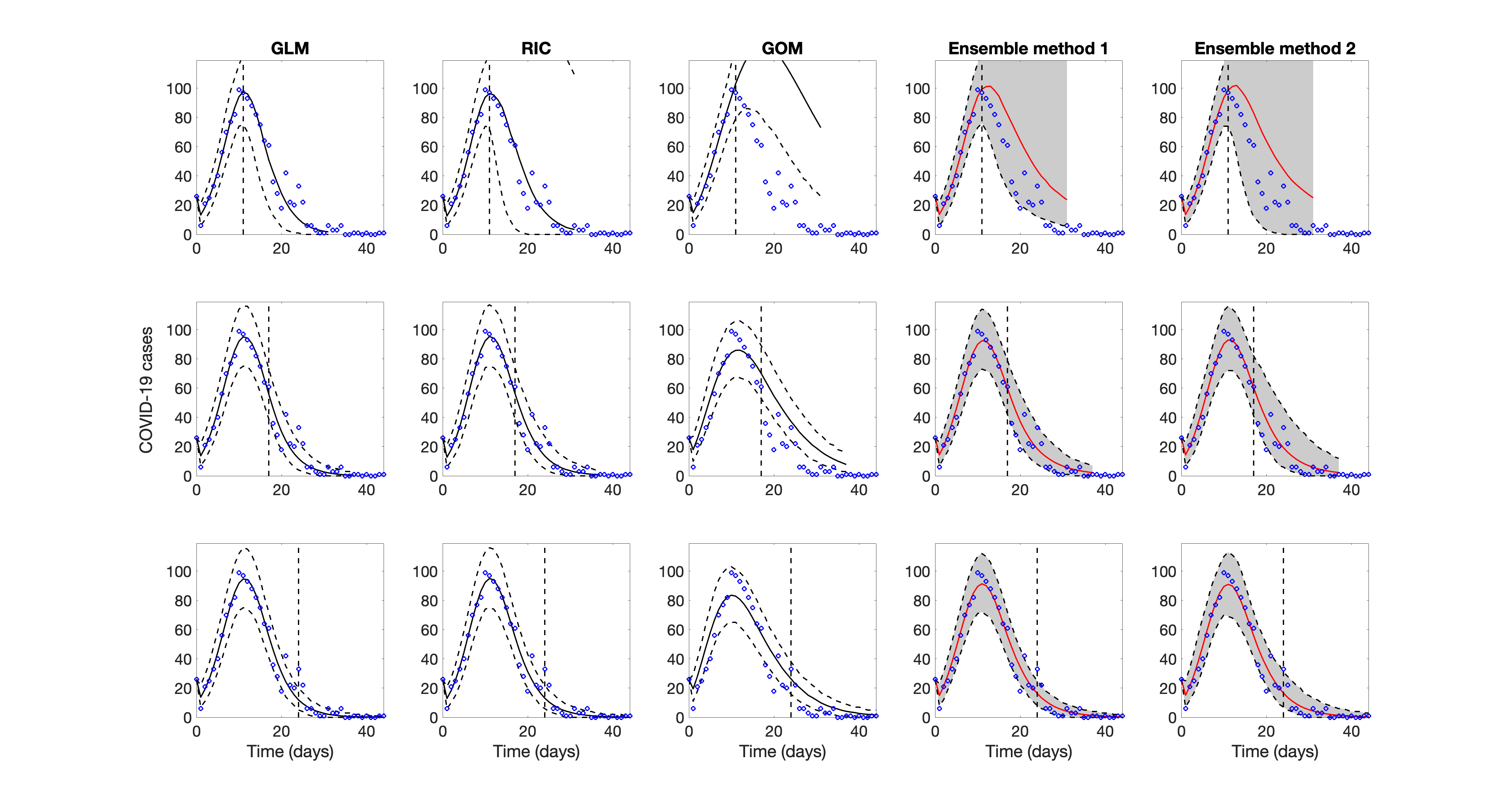


**Figure S17**. Representative sequential 20-day ahead forecasts (top to bottom panels) obtained from individual models (GLM, RIC, GOM) and two ensemble methods applied to **the COVID-19 epidemic in Henan.** Blue circles correspond to the data points. The mean fit (solid line) and 95% prediction interval (dashed lines) are also shown. The gray shaded areas further highlight differences in the 95% prediction intervals associated with the ensemble methods. The vertical line separates the calibration period (left) from the forecasting period (right).


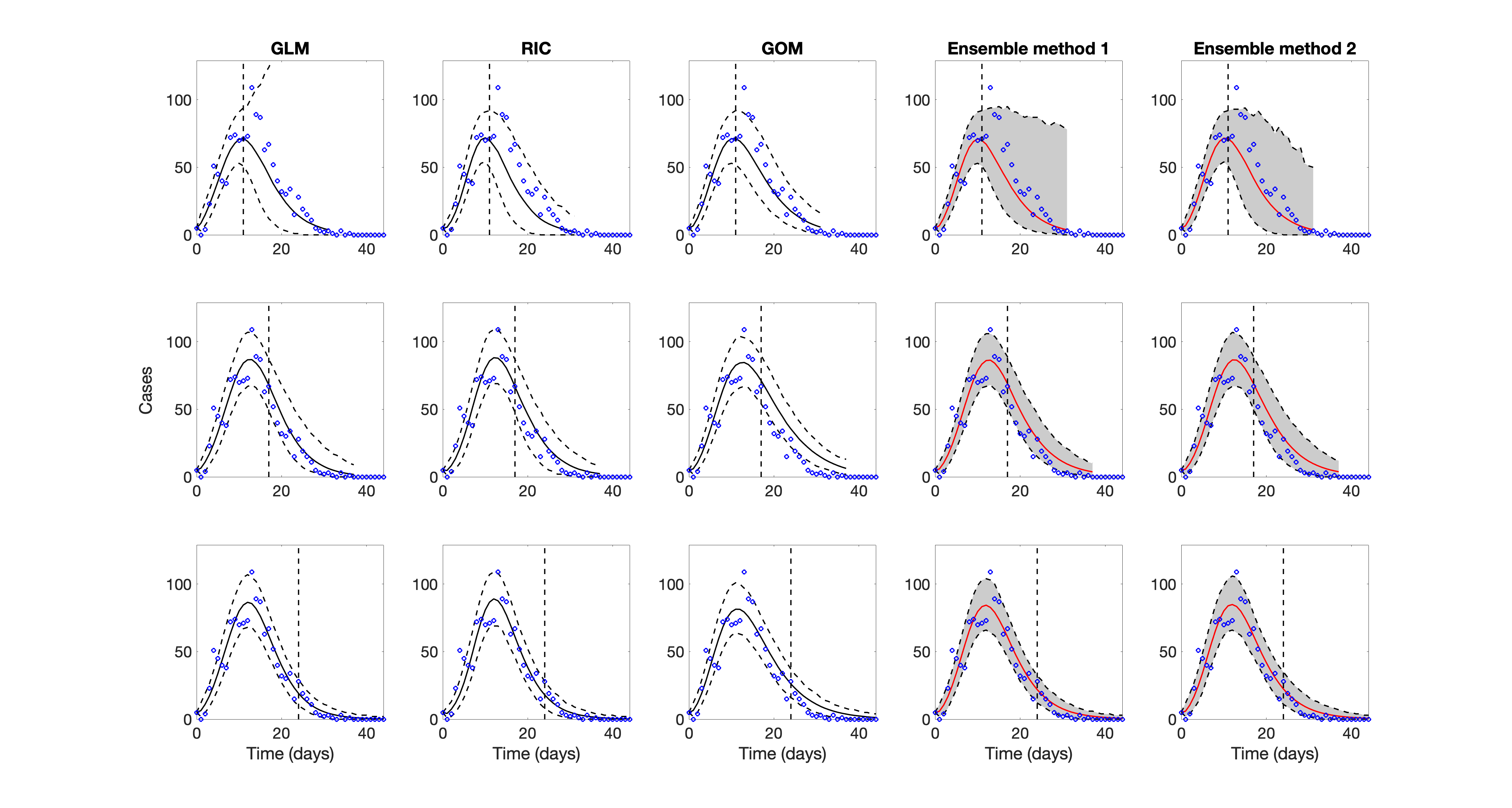


**Figure S18**. Representative sequential 20-day ahead forecasts (top to bottom to panels) obtained from individual models (GLM, RIC, GOM) and two ensemble methods applied to **the COVID-19 epidemic in Hunan.** Blue circles correspond to the data points. The mean fit (solid line) and 95% prediction interval (dashed lines) are also shown. The gray shaded areas further highlight differences in the 95% prediction intervals associated with the ensemble methods. The vertical line separates the calibration period (left) from the forecasting period (right).


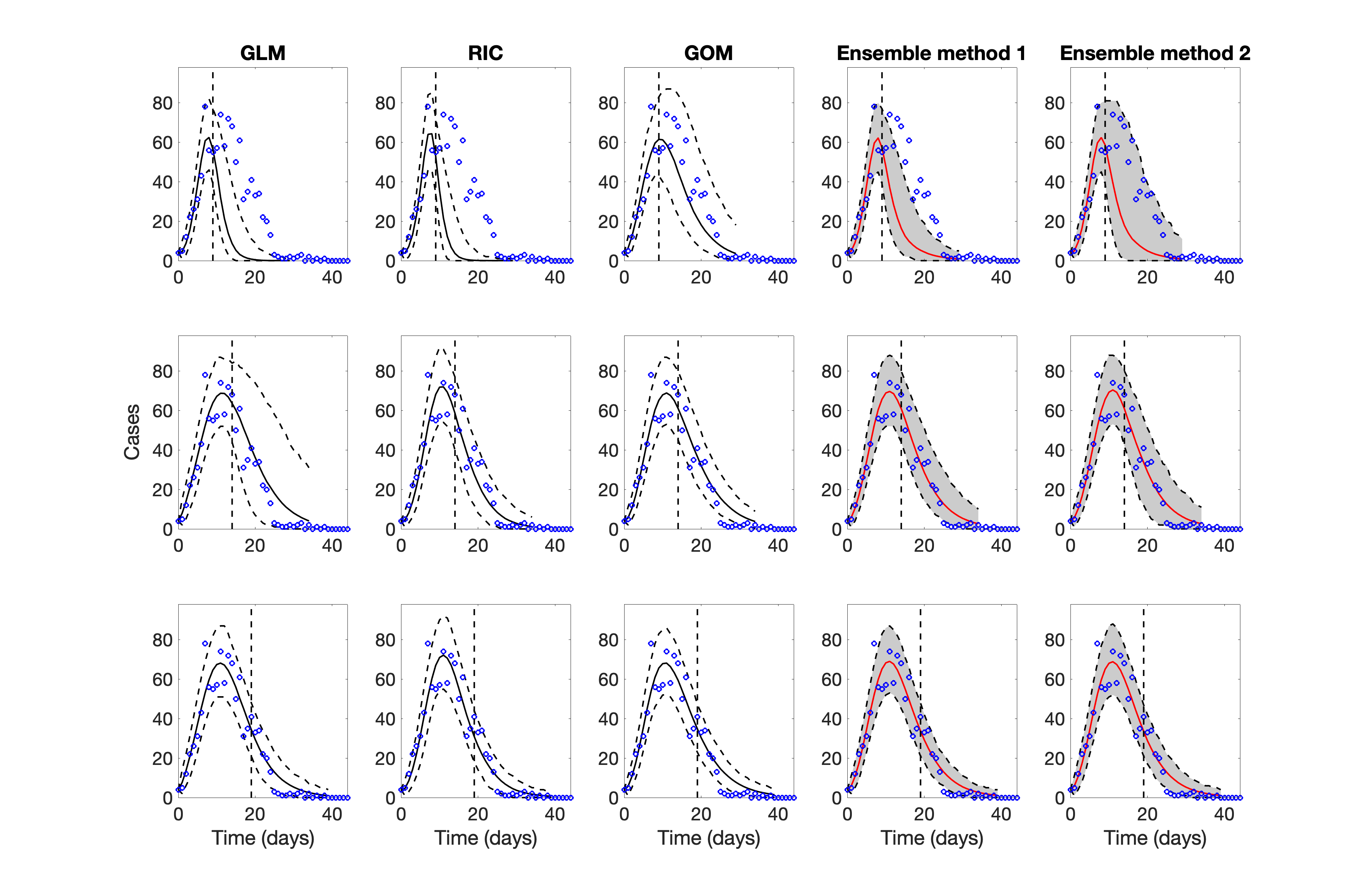


**Figure S19**. Representative sequential 20-day ahead forecasts (top to bottom panels) obtained from individual models (GLM, RIC, GOM) and two ensemble methods applied to **the Zika epidemic in Antioquia, Colombia.** Blue circles correspond to the data points. The mean fit (solid line) and 95% prediction interval (dashed lines) are also shown. The gray shaded areas further highlight differences in the 95% prediction intervals associated with the ensemble methods. The vertical line separates the calibration period (left) from the forecasting period (right).


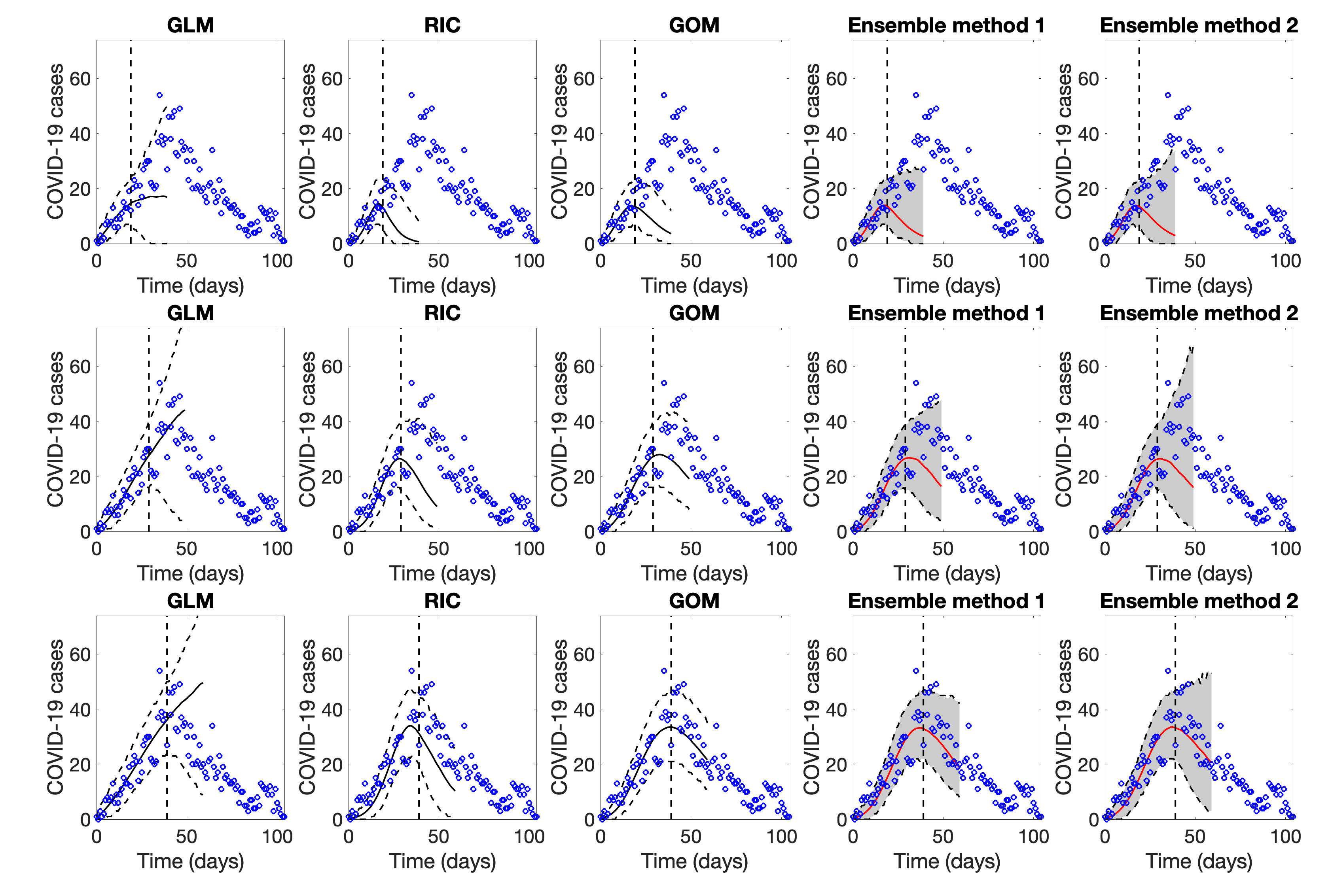

Supplement: Supplementary file 1 — Additional file 1: Figure S1. Weekly incidence curves of the four epidemic scenarios of the Ebola Forecasting Challenge (blue circles). The dashed vertical lines indicate the start and end weeks of the weekly 4-week ahead forecasts. Figure S2. Representative sequential 20-day ahead forecasts (top to bottom panels) obtained from individual models (GLM, RIC, GOM) and two ensemble methods applied to synthetic data derived from a stochastic SEIR model with a population size of 100,000 and a time-dependent transmission rate (Fig. 3). Blue circles correspond to the data points. The mean fit (solid line) and 95% prediction interval (dashed lines) are also shown. The gray shaded areas help highlight differences in the 95% prediction intervals for the two ensemble methods. The vertical line separates the calibration period (left) from the forecasting period (right). Figure S3. Mean performance of the individual models and ensemble models in 1–20 day ahead forecasts from the synthetic data derived from the stochastic SEIR model with time-dependent transmission rate (Fig. 3). Our findings indicate that the Ensemble Method 2 outperformed all other models including Ensemble Method 1 based on the coverage rate of the 95% PI, which was closer to 0.95, and the MIS. Although the RIC model achieved a lower MAE and MSE at longer horizons compared to both Ensemble Methods, Ensemble Method 2 outperformed the other models including the Ensemble Method 1 based on the coverage rate and the MIS. Figure S4. Representative sequential 20-day ahead forecasts (top to bottom panels) obtained from individual models (GLM, RIC, GOM) and two ensemble methods applied to Scenario 1 of the Ebola Forecasting Challenge (Figure S1). Blue circles correspond to the data points. The mean fit (solid line) and 95% prediction interval (dashed lines) are also shown. The gray shaded areas further highlight differences in the 95% prediction intervals associated with the ensemble methods. The vertical line separates [file 12874_2021_1226_MOESM1_ESM.docx]
